# Adiposity and risk of breast cancer in 280 000 Chinese women: a prospective cohort study

**DOI:** 10.1093/ije/dyag155

**Published:** 2026-08-19

**Authors:** Christiana Kartsonaki, Neil Wright, Iona Millwood, Yuanjie Pang, Dianjianyi Sun, Pei Pei, Dan Schmidt, Rebecca Stevens, Yuan Cao, Huaidong Du, Jun Lv, Yiping Chen, Canqing Yu, Junshi Chen, Derrick Bennett, Liming Li, Ling Yang, Zhengming Chen

**Affiliations:** Clinical Trial Service Unit & Epidemiological Studies Unit (CTSU), Nuffield Department of Population Health, Big Data Institute Building, University of Oxford, Oxford, United Kingdom; Clinical Trial Service Unit & Epidemiological Studies Unit (CTSU), Nuffield Department of Population Health, Big Data Institute Building, University of Oxford, Oxford, United Kingdom; Clinical Trial Service Unit & Epidemiological Studies Unit (CTSU), Nuffield Department of Population Health, Big Data Institute Building, University of Oxford, Oxford, United Kingdom; Department of Epidemiology and Biostatistics, School of Public Health, Peking University Health Science Center, Beijing, China; Peking University Center for Public Health and Epidemic Preparedness & Response, Beijing, China; Key Laboratory of Epidemiology of Major Diseases (Peking University), Ministry of Education, Beijing, China; Department of Epidemiology and Biostatistics, School of Public Health, Peking University Health Science Center, Beijing, China; Peking University Center for Public Health and Epidemic Preparedness & Response, Beijing, China; Key Laboratory of Epidemiology of Major Diseases (Peking University), Ministry of Education, Beijing, China; Peking University Center for Public Health and Epidemic Preparedness & Response, Beijing, China; Clinical Trial Service Unit & Epidemiological Studies Unit (CTSU), Nuffield Department of Population Health, Big Data Institute Building, University of Oxford, Oxford, United Kingdom; Clinical Trial Service Unit & Epidemiological Studies Unit (CTSU), Nuffield Department of Population Health, Big Data Institute Building, University of Oxford, Oxford, United Kingdom; Tongxiang CDC, Tongxiang, Zhejiang, China; Clinical Trial Service Unit & Epidemiological Studies Unit (CTSU), Nuffield Department of Population Health, Big Data Institute Building, University of Oxford, Oxford, United Kingdom; Department of Epidemiology and Biostatistics, School of Public Health, Peking University Health Science Center, Beijing, China; Peking University Center for Public Health and Epidemic Preparedness & Response, Beijing, China; Key Laboratory of Epidemiology of Major Diseases (Peking University), Ministry of Education, Beijing, China; Clinical Trial Service Unit & Epidemiological Studies Unit (CTSU), Nuffield Department of Population Health, Big Data Institute Building, University of Oxford, Oxford, United Kingdom; Department of Epidemiology and Biostatistics, School of Public Health, Peking University Health Science Center, Beijing, China; Peking University Center for Public Health and Epidemic Preparedness & Response, Beijing, China; Key Laboratory of Epidemiology of Major Diseases (Peking University), Ministry of Education, Beijing, China; China National Center for Food Safety Risk Assessment, Beijing, P. R. China; Clinical Trial Service Unit & Epidemiological Studies Unit (CTSU), Nuffield Department of Population Health, Big Data Institute Building, University of Oxford, Oxford, United Kingdom; Department of Epidemiology and Biostatistics, School of Public Health, Peking University Health Science Center, Beijing, China; Peking University Center for Public Health and Epidemic Preparedness & Response, Beijing, China; Key Laboratory of Epidemiology of Major Diseases (Peking University), Ministry of Education, Beijing, China; Clinical Trial Service Unit & Epidemiological Studies Unit (CTSU), Nuffield Department of Population Health, Big Data Institute Building, University of Oxford, Oxford, United Kingdom; Clinical Trial Service Unit & Epidemiological Studies Unit (CTSU), Nuffield Department of Population Health, Big Data Institute Building, University of Oxford, Oxford, United Kingdom

**Keywords:** adiposity, breast cancer, China

## Abstract

**Background:**

Adiposity is associated with increased risk of breast cancer in post-menopausal women, while the association appears to be the inverse among premenopausal women in Western populations, but is unclear for Chinese women.

**Methods:**

Using data on 284 538 women with no history of cancer, hysterectomy, oophorectomy, or breast surgery at baseline in 2004–8 from the China Kadoorie Biobank (a prospective cohort study), Cox regression was used to estimate adjusted hazard ratios (HRs) for breast cancer and its subtypes by measured body mass index (BMI) and other adiposity measures (waist circumference, fat percentage, waist–hip ratio, fat mass) and by self-reported BMI at age 25 years.

**Results:**

Mean BMI was 23.8 (3.5 SD) kg/m^2^ at baseline (mean age 51.4 years) and 21.9 (2.7 SD) kg/m^2^ at age 25 years. During 10 years of follow-up, there were 2379 incident breast cancer cases. Higher BMI was associated with a higher risk of breast cancer in women who were post-menopausal [HR = 1.35, 95% confidence interval (CI) 1.24–1.47] and premenopausal (HR = 1.13, 95% CI 1.03–1.24) at baseline, but not when censoring at age 50 years to capture mainly premenopausal cancers. Among post-menopausal women, BMI was associated with oestrogen-receptor positive (ER ) (HR = 1.47, 95% CI 1.24–1.74), but not with oestrogen-receptor negative (ER−) breast cancer (HR = 1.02, 95% CI 0.75–1.37). Waist circumference and fat percentage were associated with a higher risk of breast cancer.

**Conclusion:**

In this cohort of Chinese women, higher levels of general and central adiposity were associated with a higher risk of breast cancer, in both women who were premenopausal and women who were post-menopausal at baseline. Among post-menopausal women, adiposity was associated with ER   but not ER − breast cancer.

Key Messages

- We investigated the association of adiposity with risk of breast cancer and its subtypes in Chinese pre- and post-menopausal women in a prospective cohort study of ∼280 000 women.
- We found that body mass index and waist circumference measured in pre- and post-menopausal women are associated with oestrogen-receptor positive (ER ) but not negative breast cancer, but not when restricting to cancers that occurred before age 50 years.
- General and central adiposity are associated with risk of post-menopausal ER   breast cancer in Chinese women, similarly to other populations, but with no evidence of an inverse association with premenopausal breast cancer.

## Introduction

Breast cancer is the most common cancer among women worldwide and in China, where 268 600 new cases of breast cancer were estimated to have been diagnosed in 2015 [1], and the incidence has been increasing over the past decades [2]. Although mortality due to breast cancer has decreased mainly due to incremental advances in treatment during the last few decades, breast cancer still is a major cause of cancer-related mortality worldwide and the leading cause of cancer death in women, with 685 000 deaths globally in 2020 [3]. In China, the mean age at diagnosis of breast cancer in women is lower than those in Western populations [4]. The distribution of subtypes and molecular features of breast cancer is different in China compared with those in Western women, with HER2-enriched subtypes being more frequent than in other populations [5–7] and with patients having different responses to certain treatments. Moreover, adiposity (both general adiposity levels and fat distribution) and reproductive characteristics (parity, age at menarche and menopause) [8, 9] as well as mammographic density differ between Western and Chinese women [10].

Both in China and worldwide, levels of adiposity have been increasing [11]. Adiposity is associated with an increased risk of breast cancer among post-menopausal women in Western populations [12, 13]. However the association of adiposity with risk of breast cancer appears to be the inverse among premenopausal women [14], although this finding is not ubiquitous among studies [15, 16] and has been shown to vary by ethnicity, with studies on Asian women showing an association with a higher risk among premenopausal women [17, 18]. This may be partly due to the relationship of adiposity with specific breast cancer subtypes, which have distinct aetiologies [19]. It has been reported that obesity is associated with a higher risk of triple-negative [oestrogen-receptor (ER), progesterone-receptor (PR), and HER2 negative] breast cancer, particularly among premenopausal women [20], but there is still some uncertainty about the association of adiposity with ER negative (ER−) breast cancer, as other studies have found no association with ER − disease [21] or only in certain subsets of participants [22]. In general, there is uncertainty about the associations of adiposity with subtypes defined by tumour features [23]. Also, the association of body size over the life course with post-menopausal breast cancer varies from inverse (in early life) to positive (after menopause) [24] and long-term weight gain is associated with a higher risk of post-menopausal breast cancer [25].

Moreover, uncertainty remains about the role of features describing body size other than body mass index (BMI), which may reflect different underlying aetiological factors of body composition [26]. There has been some evidence that the waist–hip ratio is associated with a higher breast cancer risk among pre- and post-menopausal women [27, 28]. However, studies on body composition and the risk of breast cancer are primarily in Western populations and there is limited evidence on potential differences between ethnicities.

We examined the associations of general (BMI, young-adulthood BMI, body fat percentage, fat mass) and central (waist circumference, waist–hip ratio) adiposity with breast cancer in the China Kadoorie Biobank (CKB)—a prospective cohort study including ∼280 000 women. We also assessed the associations of adiposity with molecular (ER, PR, and HER2 status) and histological (ductal and non-ductal) subtypes of breast cancer.

## Methods

### Study population and data collection

Details of the CKB design, survey methods, and population characteristics have been described elsewhere [29]. Briefly, 512 726 participants (210 204 men and 302 522 women) aged 30–79 years were recruited into the study from 1175 local communities in 10 diverse regions (5 urban and 5 rural) in China between 2004 and 2008. Prior international, national, and regional ethical approvals were obtained and all participants provided written informed consent. At study clinics, participants completed an interviewer-administered laptop-based questionnaire. A range of physical measurements were recorded by trained technicians using calibrated instruments with standard protocols.

### Assessment of adiposity

All measurements were made once by trained technicians while participants were wearing light clothes and no shoes. Standing height was measured to the nearest 0.1 cm by using a stadiometer. Weight was measured to the nearest 0.1 kg by using a body composition analyser (TANITA-TBF-300GS; Tanita Corporation), with subtraction for the weight of clothing by 0.5 kg in summer, 1.0 kg in spring/autumn, and 2.0–2.5 kg in winter. Waist and hip circumferences were measured to the nearest 0.1 cm by using a soft non-stretchable tape. Hip circumference was measured at the maximum circumference around the buttocks. The waist–hip ratio was the ratio of the waist circumference to the hip circumference. Body fat percentage was the fraction of total weight that was estimated to be fat weight via the Tanita body composition analyser using proprietary algorithms.

Adulthood BMI was calculated as the measured weight in kilograms divided by the square of the measured height in metres. Participants were asked at baseline to report their weight at age 25 years. Young-adulthood BMI was calculated by using the recalled weight at age 25 years and the measured height at baseline. Fat mass was calculated as the body fat percentage multiplied by the weight in kilograms.

### Follow-up of participants

Information on the vital statistics of participants was obtained from local death registries checked annually against local residential records and health insurance records, and supplemented by a review of the available medical records. In deaths without recent medical attention, verbal autopsies determined probable causes. Information on health outcomes was collected through linkage to cancer registries, and via each individual’s unique national identification number, to the health insurance system, which provides almost universal coverage in the study areas. All events were coded using the International Classification of Diseases and Related Health Problems, Tenth Revision (ICD-10) by trained staff who were blinded to the baseline information. By 1 January 2019, 21 351 (7.1%) of the female participants of the full cohort had died, 3354 (1.1%) were lost to follow-up, and 17 872 (5.9%) had developed cancer, including 3108 (1.0%) with breast cancer (ICD-10 C50). Additional information on cancer clinical diagnosis has been collected as part of an ongoing cancer validation programme (Supplementary methods).

### Statistical analysis

Women with a history of cancer, a breast lump removed, hysterectomy, or oophorectomy at baseline were excluded from the present study (Supplementary methods), leaving 284 526 women for the main analysis, 2379 of whom had breast cancer reported. Adjusted means and proportions by BMI were calculated by using direct standardization to the age (in 10-year age groups) and region (10 regions) structure of the CKB cohort.

Cox proportional hazards models were used to estimate adjusted hazard ratios (HRs) for breast cancer by measured BMI and other adiposity measures (waist circumference, body fat percentage, waist–hip ratio, fat mass) and self-reported BMI at age 25 years. Exposures were modelled both as continuous variables and as categorical [for BMI, five categories: underweight (<18.5 kg/m^2^), lower normal weight (18.5 to <22.5 kg/m^2^; reference), higher normal weight (22.5–<25 kg/m^2^), overweight (25 to <30 kg/m^2^), and obese (≥30 kg/m^2^); all other exposures were split at their quintiles]. Models were adjusted for age (numeric), education (no formal education/primary school, middle/high school, or technical school/university), smoking (never, occasional, ex-regular, or current regular), and alcohol consumption (never regular, ex-regular, or current regular), and stratified by region (10 regions) (i.e. a model with region-specific baseline hazards). Analyses with additional adjustment for family history of cancer [no, yes (at least one first-degree relative with a history of cancer)], use of oral contraceptives (never, past, or current), parity (number of live births, numeric), and age of menarche (numeric) were also performed. An additional analysis also adjusting for physical activity, measured by metabolic equivalent of task (MET)-hours/week, was performed. For analyses involving a categorical exposure with more than two categories, all HRs are presented with ‘floating’ standard errors to facilitate comparisons between groups [30]. Analyses were performed separately for women who were pre- and post-menopausal at baseline (excluding perimenopausal women from these analyses).

Moreover, in order to study the association of adiposity with premenopausal breast cancer separately, we fitted models to all women who were premenopausal at baseline, censoring at ages 50 and 48 years if breast cancer had not been diagnosed by then. We also performed analyses stratified by age at baseline. To estimate associations of adiposity measures with the risk of breast cancer subtypes, we censored individuals at diagnosis of a different subtype. We carried out a sensitivity analysis for ER   including only clear positives. We also estimated associations with risk of death due to breast cancer.

The proportional-hazards assumption was assessed by using scaled Schoenfeld residuals [31]. We explored the main associations in subgroups defined by age at baseline (five groups), rural/urban residence, education (three categories), smoking status (never/ever regular smoking), alcohol drinking (never/ever regular), family history of cancer (no/yes), use of oral contraceptives (never/ever), and parity (nulliparous/parous), adjusting for age and menopause status (including perimenopausal women as a separate category) and stratifying by region.

R version 4.2.2 was used for analysis [32] with packages ‘Epi’ [30], ‘DirectStandardisation’ [33], and ‘ckbplotr’ [34].

## Results

Among the 284 538 women included, the mean BMI was 23.8 (3.5 SD) kg/m^2^ at baseline (mean age 51.4 years) and 21.9 (2.7 SD) kg/m^2^ at age 25 years among 229 972 women with available data. At baseline, 126 054 women were premenopausal, 14 432 were perimenopausal, and 144 052 were post-menopausal. BMI and body fat percentage were higher on average among women aged 50–59 years at baseline compared with younger and older women, while waist circumference increased with age. Women who resided in urban regions had on average a higher BMI and those with a higher educational attainment were less likely to be overweight or obese. Women with a higher BMI had on average a lower age at menarche and were more likely to have used oral contraceptives. Underweight women were more likely to have smoked regularly. There were positive associations of BMI with the other adiposity measures (Table 1).

**Table 1:** Selected participant characteristics by BMI at study baseline.

	BMI (kg/m²)			
	<18.5	**18**–**<25**	**25**–**<30**	≥30
	(*N* = 12 395)	(*N* = 175 830)	(*N* = 82 741)	(*N* = 13 571)
Age at baseline (years)	51.4 (0.032)	51.3 (0.007)	51.4 (0.009)	51.5 (0.026)
Region				
Rural	66.7	58.6	51.9	48.4
Urban	33.3	41.4	48.1	51.6
Education				
No formal/primary school	57.1	55.8	58.7	61.7
Middle/high school	38.7	39.4	37.8	36.0
College/university	4.3	4.8	3.5	2.2
Ever regular smoker	5.3	3.3	2.9	3.5
Ever regular alcohol drinking	2.4	2.5	2.5	2.2
Physical activity (MET-h/day)	20.5 (0.12)	20.8 (0.03)	20.1 (0.04)	19.1 (0.11)
Family history of cancer	14.6	16.1	16.8	16.4
Age at menarche	15.9 (0.023)	15.5 (0.005)	15.3 (0.007)	15.0 (0.018)
Number of live births	2.2 (0.009)	2.2 (0.002)	2.3 (0.003)	2.3 (0.009)
Nulliparous	0.5	0.4	0.3	0.6
Ever breastfed^a^	94.7	96	96.3	95.5
Use of oral contraceptives	8.3	9.5	10.1	10.6
Menopause status				
Premenopausal	41.4	44.4	44.5	44
Perimenopausal	4.0	4.8	5.6	6.3
Post-menopausal	54.6	50.8	50.0	49.7
Age at menopause^b^	41.8 (0.1)	45.5 (0.1)	44.7 (0.2)	44.0 (0.3)
BMI (kg/m²)	17.5 (0.009)	22.2 (0.004)	26.8 (0.005)	31.9 (0.020)
BMI at age 25 years (kg/m²)	20.0 (0.028)	21.5 (0.007)	22.8 (0.012)	24.0 (0.039)
Waist circumference (cm)	64.3 (0.05)	75.4 (0.01)	86.1 (0.02)	96.7 (0.07)
Waist–hip ratio	0.79 (0.00065)	0.85 (0.00014)	0.90 (0.00021)	0.94 (0.00063)
Body fat percentage	19.2 (0.03)	29.2 (0.01)	37.9 (0.02)	45.3 (0.06)

Values are means (standard errors) or percentages, adjusted for age and region (except age, which is only adjusted for region, and region, which is only adjusted for age).

a Among parous women.

b Among post-menopausal women.

The incidence rates of breast cancer varied by age (Supplementary Table 2), with the highest standardized incidence rate at ages 50–59 years. The incidence was higher in urban areas than in rural areas (Supplementary Figure 1). Among women with available data, cancers were most frequently Stage 2, ER positive (ER ), and Grade 2 (Table 2 and Supplementary Table 1).

**Table 2:** Selected tumour characteristics of breast cancer cases by age at diagnosis. Numbers of cases and percentages by age are among the 284 526 women included in the main analysis. Percentages are calculated among women with available data for each characteristic.

	Age at diagnosis (years)				
	30–44	45–54	55–69	≥70	Total
Total *N* (%)	229 (9.6)	825 (34.7)	1030 (43.3)	295 (12.4)	2379
Grade					
1 (well differentiated)	1 (20.0)	4 (10.0)	4 (9.8)	1 (10.0)	10 (10.4)
2 (moderately differentiated)	3 (60.0)	19 (47.5)	23 (56.1)	6 (60.0)	51 (53.1)
3 (poorly differentiated)	1 (20.0)	17 (42.5)	14 (34.1)	3 (30.0)	35 (36.5)
Histology					
Ductal carcinoma	48 (84.2)	234 (75.0)	342 (77.7)	75 (71.4)	699 (76.5)
Lobular carcinoma	3 (5.3)	16 (5.1)	14 (3.2)	3 (2.9)	36 (3.9)
Other	6 (10.5)	62 (19.9)	84 (19.1)	27 (25.7)	179 (19.6)
Stage					
1	3 (23.1)	20 (22.7)	39 (24.8)	4 (22.2)	66 (23.9)
2	7 (53.8)	55 (62.5)	72 (45.9)	9 (50.0)	143 (51.8)
3	3 (23.1)	13 (14.8)	38 (24.2)	5 (27.8)	59 (21.4)
4			8 (5.1)		8 (2.9)
ER status					
−	10 (20.0)	90 (35.7)	112 (29.4)	18 (19.8)	230 (29.7)
	40 (80.0)	162 (64.3)	269 (70.6)	73 (80.2)	544 (70.3)
PR status					
−	11 (22.4)	113 (44.8)	154 (40.3)	21 (23.3)	299 (38.7)
	38 (77.6)	139 (55.2)	228 (59.7)	69 (76.7)	474 (61.3)
HER2 status					
−	19 (48.7)	102 (51.3)	160 (55.4)	49 (67.1)	330 (55.0)
	20 (51.3)	97 (48.7)	129 (44.6)	24 (32.9)	270 (45.0)
ER, PR, HER2 status					
ER − PR − HER2−	4 (10.3)	30 (15.2)	33 (11.5)	6 (8.5)	73 (12.2)
ER − PR − HER2	4 (10.3)	31 (15.7)	44 (15.3)	4 (5.6)	83 (13.9)
ER /PR HER2−	15 (38.5)	71 (35.9)	126 (43.8)	41 (57.7)	253 (42.4)
ER /PR HER2	16 (41.0)	66 (33.3)	85 (29.5)	20 (28.2)	187 (31.4)

**Figure 1:**
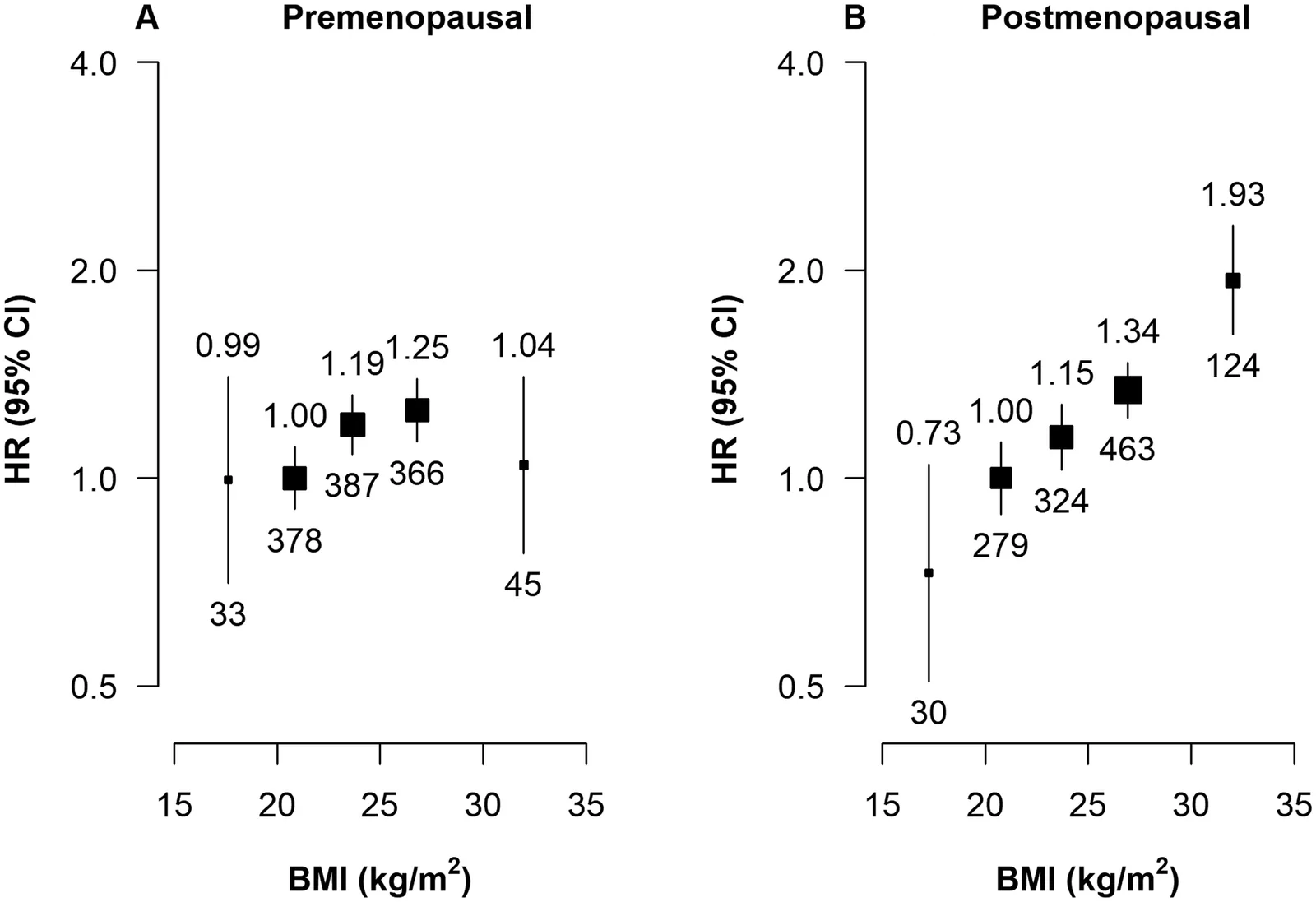
HRs (95% CIs) for breast cancer by BMI categories among women who were (a) premenopausal and (b) post-menopausal at baseline. Models are adjusted for age (numeric), smoking status (never, occasional, ex-regular, or current regular), alcohol drinking (never regular, ex-regular, or current regular), and education (no formal/primary, middle/high school, or technical school/university), and stratified by region. The numbers above the points are HRs and the numbers below the points are numbers of cases. BMI categories: underweight (<18.5 kg/m^2^), lower normal weight (18.5 to <22.5 kg/m^2^; reference), higher normal weight (22.5 to <25 kg/m^2^), overweight (25 to <30 kg/m^2^), and obese (≥30 kg/m^2^). Points are plotted at the mean of the category along the horizontal axis. 95% CIs are calculated by using ‘floating’ standard errors.

**Figure 2:**
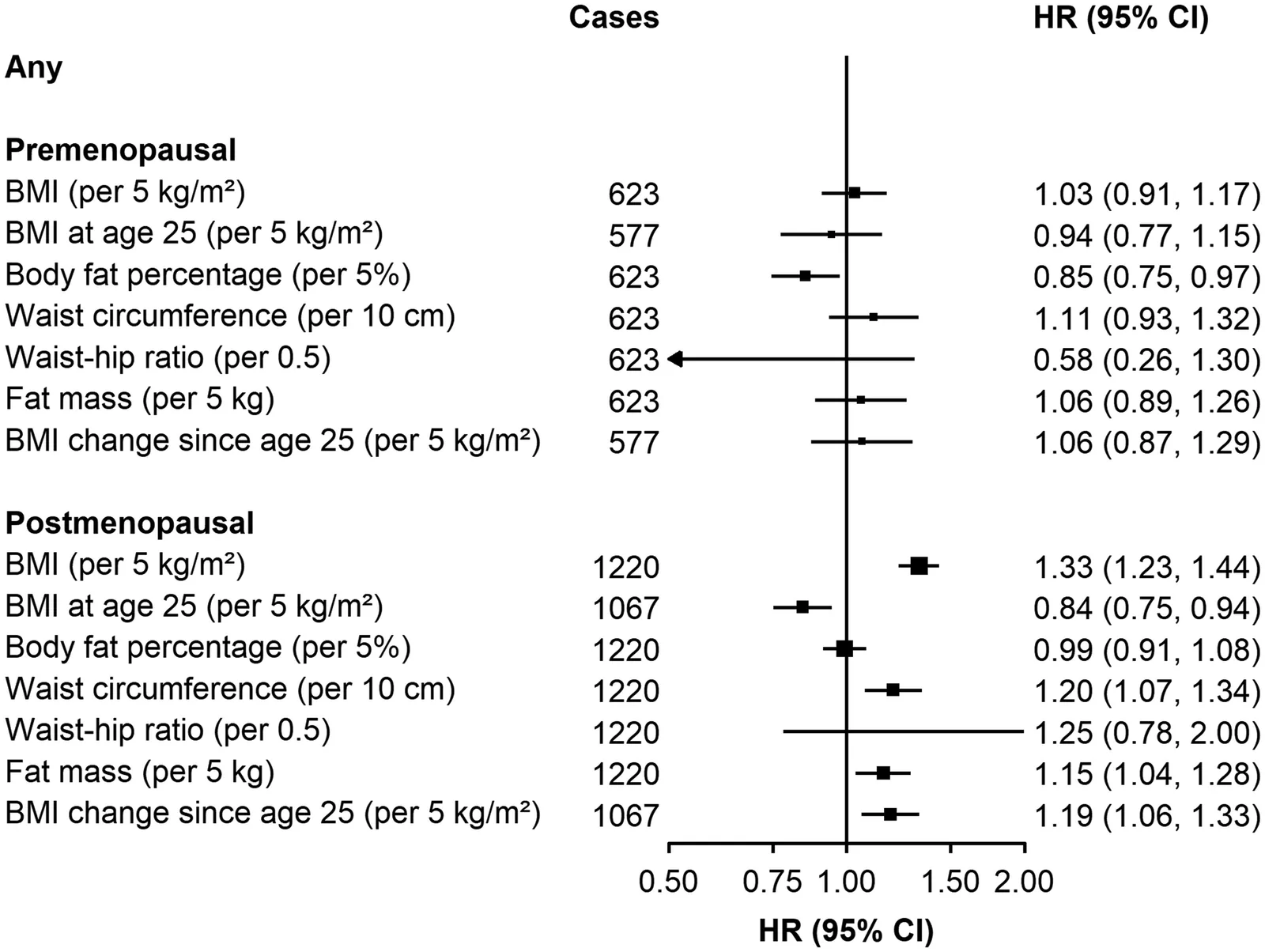
HRs (95% CIs) for breast cancer by each adiposity measure, adjusting for BMI, among women who were pre- and post-menopausal at baseline, censoring premenopausal women at age 50 years. Models are adjusted for age (numeric), smoking status (never, occasional, ex-regular, or current regular), alcohol drinking (never regular, ex-regular, or current regular), education (no formal/primary, middle/high school, or technical school/university), and BMI (numeric), and stratified by region.

Higher BMI was associated with a higher risk of breast cancer among both women who at baseline were premenopausal [adjusted HR = 1.15, 95% confidence interval (CI) 1.05–1.26, per 5 kg/m^2^ higher BMI] and women who at baseline were post-menopausal (HR = 1.33, 95% CI 1.23–1.44) (Supplementary Figure 2), with associations consistent with a log-linear form (Fig. 1). Higher waist circumference was associated with a higher risk (HR per 10-cm increase = 1.14, 95% CI 1.07–1.22 among premenopausal women at baseline and HR per 10-cm increase = 1.26, 95% CI 1.19–1.34 among post-menopausal women), which persisted after adjustment for BMI (HR per 10-cm increase = 1.16, 95% CI 1.02–1.32 and HR per 10-cm increase = 1.20, 95% CI 1.07–1.34, respectively). A higher body fat percentage was associated with a higher risk (HR per 5% increase = 1.06, 95% CI 1.01–1.10 and HR per 5% increase = 1.13, 95% CI 1.09–1.17), but not when adjusting for BMI (HR per 5% increase = 0.98, 95% CI 0.89–1.08 and HR per 5% increase = 0.98, 95% CI 0.90–1.07) in pre- and post-menopausal women, respectively (Supplementary Figure 2). When both BMI and waist circumference were included in a model, among post-menopausal women, the HRs were 1.08 (95% CI 0.93–1.25) and 1.20 (95% CI 1.07–1.34) for BMI (per 5 kg/m^2^ higher) and waist circumference (per 10 cm higher), respectively (Supplementary Figures 2 and 3). There was no evidence of an association of waist–hip ratio with risk of breast cancer. Higher fat mass was associated with a higher risk among post-menopausal women (HR = 1.16, 95% CI 1.12–1.20 per 5 kg), also when adjusting for BMI (HR = 1.15, 95% CI 1.04–1.28) (Supplementary Figure 2). Associations were not substantially altered following additional adjustment for family history of cancer and reproductive factors (premenopausal at baseline, HR per 5-kg/m^2^ higher BMI = 1.13, 95% CI 1.03–1.23, Supplementary Figure 4; post-menopausal at baseline, HR per 5-kg/m^2^ higher BMI = 1.32, 95% CI 1.21–1.42, Supplementary Figure 5), nor when additionally adjusted for physical activity (premenopausal at baseline, HR per 5-kg/m^2^ higher BMI = 1.12, 95% CI 1.03–1.23; post-menopausal at baseline, HR per 5-kg/m^2^ higher BMI = 1.31, 95% CI 1.21–1.42). When censoring premenopausal women at age 50 years to capture mainly cancers that occurred while women were still premenopausal, there was no evidence of associations of BMI (HR per 5-kg/m^2^ higher BMI = 1.05, 95% CI 0.90, 1.22), BMI at 25 (HR per 5-kg/m^2^ higher BMI = 0.95, 95% CI 0.75, 1.21), body fat percentage (HR per 5-kg/m^2^ higher BMI = 0.88, 95% CI 0.75, 1.03), or waist circumference (HR per 5-kg/m^2^ higher BMI = 1.15, 95% CI 0.93, 1.42), adjusting for BMI (Fig. 2). Results were similar when censoring at age 48 years (Supplementary Figure 6).

**Figure 3:**
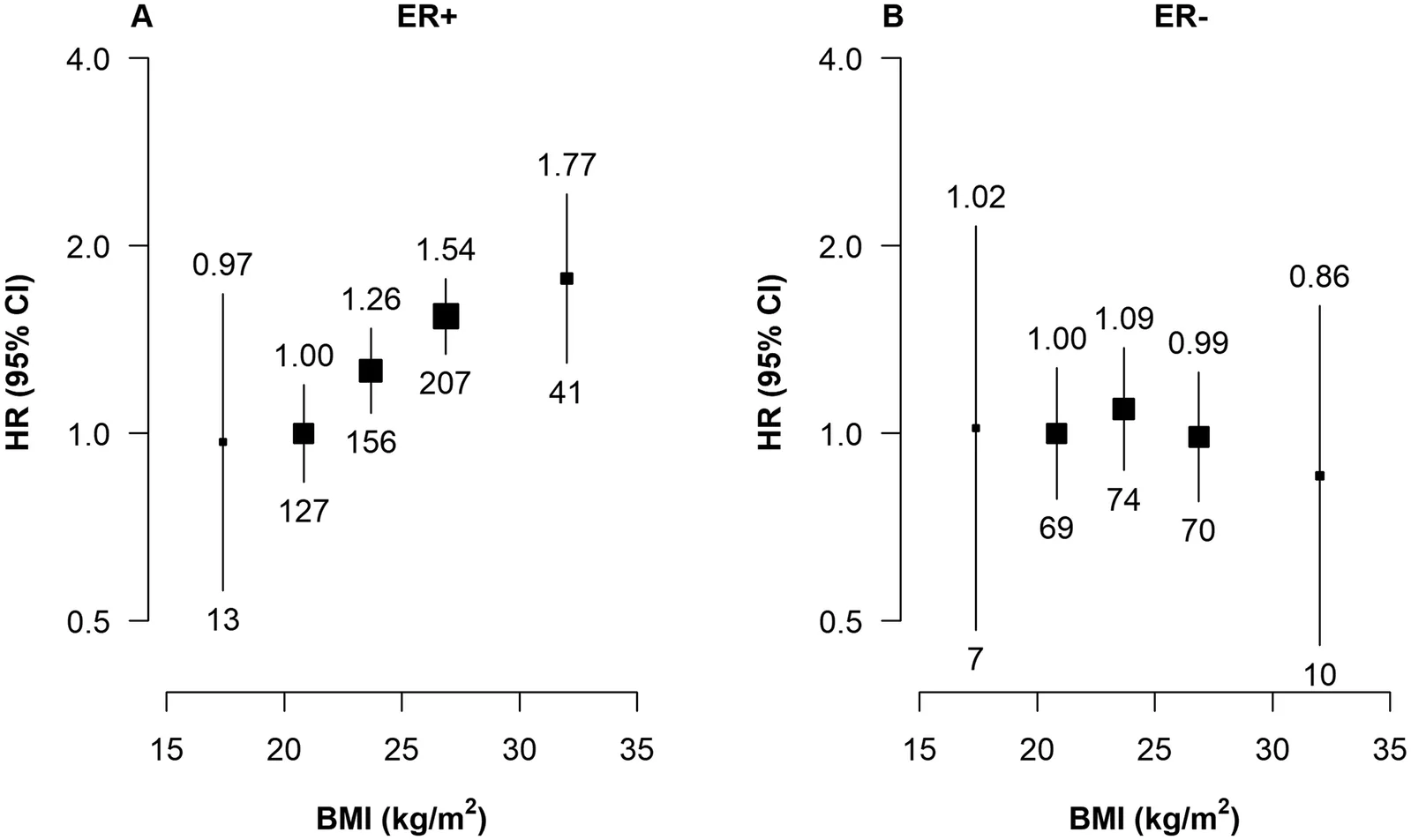
HRs (95% CIs) for (a) ER   and (b) ER − breast cancer by BMI categories among women who were post-menopausal at baseline. Models are adjusted for age (numeric), smoking status (never, occasional, ex-regular, or current regular), alcohol drinking (never regular, ex-regular, or current regular), and education (no formal/primary, middle/high school, or technical school/university), and stratified by region. BMI categories: underweight (<18.5 kg/m^2^), lower normal weight (18.5 to <22.5 kg/m^2^; reference), higher normal weight (22.5 to <25 kg/m^2^), overweight (25 to <30 kg/m^2^), and obese (≥30 kg/m^2^). Points are plotted at the mean of the category along the horizontal axis. The numbers above the points are HRs and the numbers below the points are numbers of cases. 95% CIs are calculated by using ‘floating’ standard errors.

**Figure 4:**
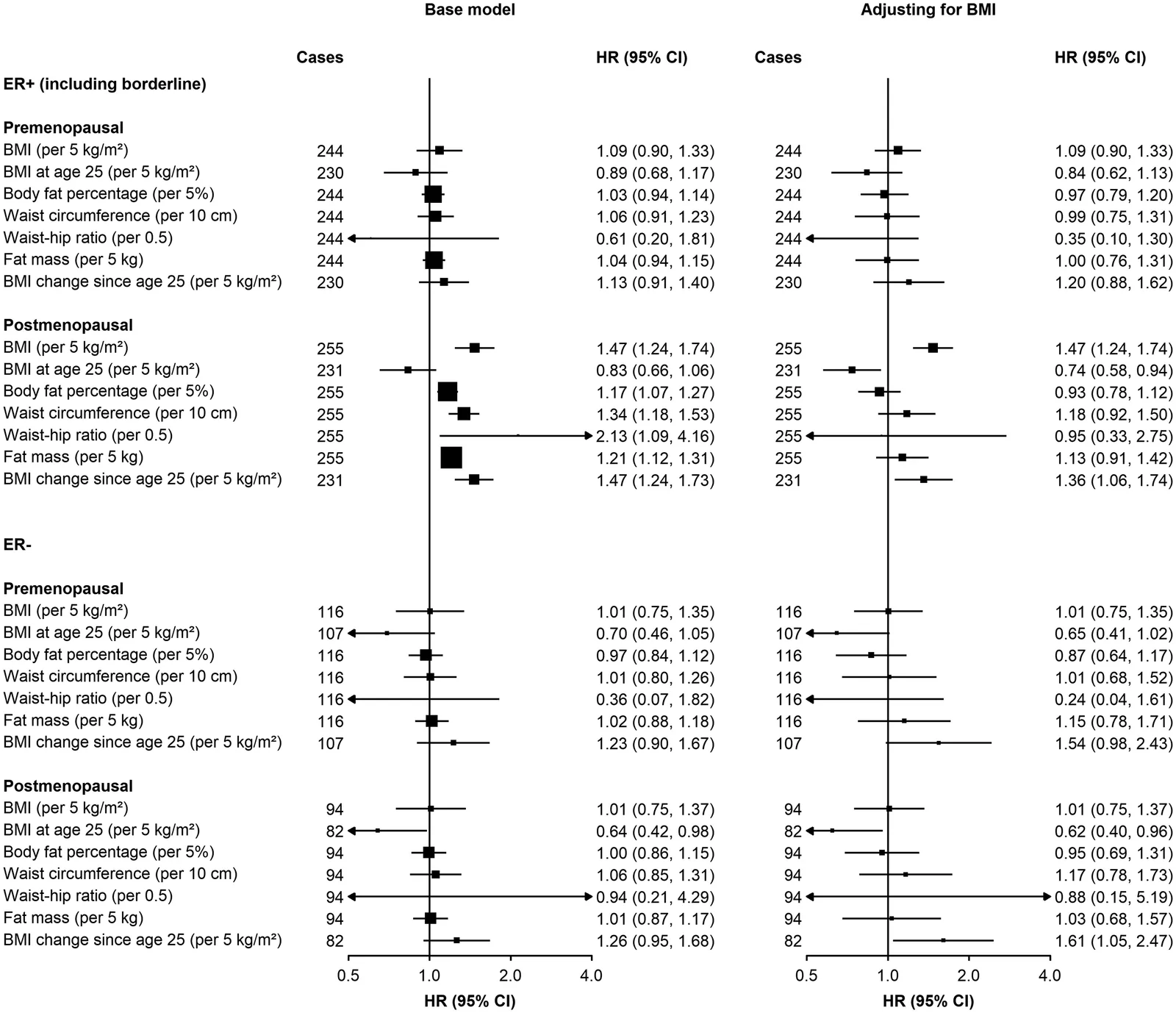
HRs (95% CIs) for ER   and ER − breast cancer by each adiposity measure among women who were pre- and post-menopausal at baseline. Models are adjusted for age (numeric), smoking status (never, occasional, ex-regular, or current regular), alcohol drinking (never regular, ex-regular, or current regular), and education (no formal/primary, middle/high school, or technical school/university), and stratified by region.

Among post-menopausal women, BMI was associated with risk of ER   with a log-linear relation (Fig. 3) (HR = 1.47, 95% CI 1.24–1.74 per 5 kg/m^2^), but not with risk of ER − breast cancer (HR = 1.01, 95% CI 0.75–1.37) (Fig. 4). Waist circumference was associated with ER (HR = 1.34, 95% CI 1.18–1.53) but not ER− (HR = 1.06, 95% CI 0.85–1.31) breast cancer among post-menopausal women. Among post-menopausal women and overall, the associations of adiposity with risk of breast cancer varied by histological subtype, with associations having greater magnitudes for lobular than for ductal tumours (Supplementary Figure 7), as lobular cancers are almost always ER ; among all women, the HR for ductal tumours was 1.21 (95% CI 1.08–1.35) and the HR for lobular tumours was 1.65 (95% CI 1.04–2.62) per 5-kg/m^2^ higher BMI. However, these results have to be interpreted with caution due to the small numbers of cases. Among women who were premenopausal at baseline, there was no evidence of an association of BMI with risk of ER (HR = 1.09, 95% CI 0.90, 1.33) or ER− (HR = 1.01, 95% CI 0.75–1.35) breast cancer (Supplementary Figure 8) or of waist circumference with either subtype (Supplementary Figure 9).

Among post-menopausal women, the associations with ER   breast cancer were similar to those observed for any breast cancer (BMI at age 25 years, HR = 0.83, 95% CI 0.66–1.06; body fat percentage, HR = 1.17, 95% CI 1.07–1.27; and waist circumference, HR = 1.34, 95% CI 1.18–1.53). There was no evidence of any associations of other adiposity measures with risk of ER   or ER − breast cancer among premenopausal women, but the numbers of events were relatively small (Fig. 4). There was no evidence of an association of BMI at age 25 years with risk of breast cancer, either in women who were premenopausal (HR per 5 kg/m^2^ = 0.92, 95% CI 0.81, 1.04, Supplementary Figures 2 and 4) or in women who were post-menopausal (HR per 5 kg/m^2^ = 0.92, 95% CI 0.83, 1.03, Supplementary Figures 2 and 5) at baseline. However, with adjustment for BMI at baseline, inverse associations of BMI at age 25 years with risk of breast cancer were observed (premenopausal at baseline, HR per 5 kg/m^2^ = 0.81, 95% CI 0.71, 0.93; post-menopausal at baseline, HR per 5 kg/m^2^ = 0.84, 95% CI 0.75–0.94).

Among post-menopausal women, BMI was associated with a higher risk of HER2 breast cancer (HR = 1.45, 95% CI 1.19, 1.77). The association of BMI with risk of HER2− cancer was weaker (HR = 1.27, 95% CI 0.94, 1.71), but consistent with that with HER2 . BMI at age 25 years was inversely associated with HER2 breast cancer among both pre- and post-menopausal women. Among premenopausal women at baseline, associations of all baseline adiposity measures with both HER2 and HER2− subtypes were null. Post-menopausal body fat percentage and waist circumference were associated with both HER2 and HER2− breast cancer, but not following additionally adjustment for BMI (Supplementary Figure 10).

The HRs of any breast cancer by BMI increased with increasing age at baseline, consistently with the associations in pre- and post-menopausal women (Supplementary Figure 11). There were no other differences between the subgroups considered, although the association was somewhat stronger among women with a lower educational attainment. There was a similar pattern for HRs by waist circumference (Supplementary Figure 12). There were 413 deaths due to breast cancer and BMI was associated with a higher risk of death due to breast cancer (HR = 1.35, 95% CI 1.17–1.54).

## Discussion

This is the first large prospective study on the association between adiposity measures and risk of breast cancer and its subtypes in China. We found an association of both pre- and post-menopausal general and central adiposity with a higher risk of breast cancer, but not when censoring premenopausal women at age 50 years to include cancers that are likely to have occurred before menopause. The associations were positive for ER   and null for ER − breast cancer. Central adiposity was associated with a higher risk also after adjustment for BMI, but the association of BMI was attenuated when waist circumference was included in the model. Adjustment for additional factors associated with risk of breast cancer did not substantially change the findings.

Our findings are consistent with results from the Million Women Study [35] in the UK, including ∼6000 cases of breast cancer, which found a ∼1.4-fold increase in risk per 10 units of BMI among post-menopausal women, but an inverse association among premenopausal women at recruitment, and with results from the IBIS trials (∼10 000 women at high risk of breast cancer and ∼800 cases) [36]. A pooled analysis of 20 prospective studies (∼1 000 000 women and ∼36 000 breast cancer cases) showed an inverse association between adiposity and risk of premenopausal breast cancer, with obese women having a relative risk of 0.78 (95% CI 0.64–0.93) compared with women with a BMI of <21 kg/m^2^ at baseline [37]. Similar associations were observed among 6 million Korean women [38] and in the Breast Cancer Surveillance Consortium (∼21 000 breast cancer cases, of whom 576 were Asian) [39]. Systematic reviews found heterogeneity of the association among premenopausal women by ethnicity [17, 27], with a positive association of BMI with breast cancer risk among Asian premenopausal women. This may be related to differences in body shape and fat percentage at a given BMI between ethnicities [40].

Adiposity is associated with poorer outcomes in breast cancer patients [41], which appears to be the case mainly for ER   tumours [42], and a Mendelian randomization study has found it to be restricted to ER   tumours [43]. We found that higher BMI at baseline was associated with a higher risk of death due to breast cancer. However, Mendelian randomization studies have found an inverse association of genetically predicted BMI with risk of breast cancer in both pre- and post-menopausal women [44–47]. This is not necessarily inconsistent with the positive associations found in observational studies, as the estimand of Mendelian randomization studies is different. Genetic associations for childhood BMI [44] are consistent with observational associations with early-life BMI [24].

The differences in the associations between adiposity during different times across the life course with risk of breast cancer before and after menopause are thought to relate to differences in oestrogen production and levels during these time periods. In post-menopausal women, oestrogen is produced primarily from fat, leading to higher oestrogen levels in overweight and obese women. In contrast, in premenopausal women, the primary source of oestrogen production is the ovaries and body size does not lead to substantial variation in oestrogen levels [48]. Other potential mechanisms include lower levels of sex hormone binding globulin with increasing adiposity, other growth factors such as insulin and insulin-like growth factor, and inflammation. The associations of features of body composition with breast cancer risk are considered to be to partly mediated by hormone levels [49] and may be modified by menopause hormone therapy (MHT) use [50].

The primary strength of the present study is the availability of weight, height, waist circumference, hip circumference, and fat percentage, measured by trained staff accurately for all ∼0.3 million women (weight at age 25 years was self-reported), as well as of information on confounders, which we adjusted for. Moreover, many of the reported events were validated by the retrieval of hospital records and we had detailed information for such validated breast cancer events, such as molecular markers, histological subtypes, grade, and stage. The study also has limitations. First, menopause status was only available at baseline for the whole cohort, so the menopause status at the time of breast cancer diagnosis was not known. However, we attempted to censor women at ages 50 and 48 years in sensitivity analyses as a proxy of menopause age. Second, we did not have information on breast density, which is associated with a substantially higher risk of breast cancer, but may be inversely associated with adiposity [51]. Last, we did not have information on MHT use, which may have a larger effect than adipose tissue on hormone concentrations [52], but this is uncommon among Chinese women (only 0.5% of women reported ever having received MHT at the second resurvey of CKB).

## Conclusion

In summary, in this cohort of Chinese women, higher levels of general and central adiposity were associated with a higher risk of breast cancer, both in women who were premenopausal and in women who were post-menopausal at baseline, but not when restricting the follow-up to age 50 years, aiming to largely capture cancers diagnosed in premenopausal women. Among post-menopausal women, adiposity was associated with ER   but not ER − breast cancer. Waist circumference and fat percentage were associated with a higher risk of breast cancer, with waist circumference remaining associated following adjustment for BMI. We found no associations for young-adulthood BMI. Further research on hormonal factors and other biomarkers may help to clarify the complex relationships between adiposity, menopause status, and breast cancer, and their differences among Chinese and Western women.

## Ethics approval

The CKB complies with all the required ethical standards for medical research on human subjects. Ethical approvals were granted and have been maintained by the relevant institutional ethical research committees in the UK and China. Informed consent was obtained from all participants included in the study. UK: Kadoorie Study of Chronic disease in China (KSCDC)—Baseline, Oxford Tropical Research Ethics Committee (OxTREC) (2005) OxTREC Ref: 025–04. CKB (a prospective study of environmental and genetic causes of premature death in 500 000 Chinese adults)—Second resurvey (2013) OxTREC Reference: 1024–13. China: Kadoorie Study of Chronic disease in China (KSCDC)—Baseline, Chinese Centre for Disease Control and Prevention. Ethical Review Committee (2004) Approval Notice 005/2004. Kadoorie Study of Chronic disease in China (KSCDC)—Second Phase, Chinese Academy of Medical sciences/Peking Union Medical College, Ethical Committee (2010) 24 December 2010.

## Supplementary Material

dyag155_Supplementary_Data

## Data Availability

The CKB is a global resource for the investigation of lifestyle, environmental, blood biochemical, and genetic factors as determinants of common diseases. The CKB study group is committed to making the cohort data available to the scientific community in China, the UK, and worldwide to advance knowledge about the causes, prevention, and treatment of disease. For detailed information on the data that are currently available to open-access users and how to apply for them, visit http://www.ckbiobank.org/site/Data Access. Researchers who are interested in obtaining the raw data from the CKB study that underline this paper should contact ckbaccess@ndph.ox.ac.uk. A research proposal will be requested to ensure that any analysis is performed by bona fide researchers and, where data are not currently available to open-access researchers, is restricted to the topic covered in this paper. This research was funded in whole, or in part, by the Wellcome Trust (grant numbers 212946/Z/18/Z, 202922/Z/16/Z, 104085/Z/14/Z, 088158/Z/09/Z). For the purpose of open access, the author has applied a CC-BY public copyright licence to any Author Accepted Manuscript version arising from this submission.

## Members of the China Kadoorie Biobank collaborative group

International Steering Committee: Junshi Chen, Zhengming Chen (PI), Rory Collins, Liming Li (PI), Jun Lv, Richard Peto, Robin Walters, Canqing Yu, Dianjianyi Sun, Derrick Bennett.

International Co-ordinating Centre, Oxford: Daniel Avery, Maxim Barnard, Derrick Bennett, Ruth Boxall, Ka Hung Chan, Yiping Chen, Zhengming Chen, Jonathan Clarke, Robert Clarke, Huaidong Du, Ahmed Edris Mohamed, Hannah Fry, Yani Huang, Prapthi Harish, Pek Kei Im, Andri Iona, Christiana Kartsonaki, Kshitij Kolhe, Hubert Lam, Kuang Lin, Iona Millwood, Sam Morris, Qunhua Nie, Sara Perez Vizan, Thu Pham, Alfred Pozarickij, Maryam Rahmati, Paul Ryder, Maruf Sarder, Dan Schmidt, Roshi Shrestha, Becky Stevens, Robin Walters, Lin Wang, Neil Wright, Ling Yang, Pang Yao.

National Co-ordinating Centre, Beijing: Liming Li, Jun Lv, Canqing Yu, Dianjianyi Sun, Yuanjie Pang, Yuting Han, Can Hou, Qingmei Xia, Chao Liu, Pei Pei, Lang Pan, Xiao Han, Honglu Bian, Xinxin Chen.

10 Regional Co-ordinating centres:

Qingdao CDC: Zengchang Pang, Ruqin Gao, Shanpeng Li, Haiping Duan, Shaojie Wang, Yongmei Liu, Ranran Du, Yajing Zang, Liang Cheng, Xiaocao Tian, Hua Zhang, Yaoming Zhai, Feng Ning, Xiaohui Sun, Feifei Li. Licang CDC: Silu Lv, Junzheng Wang, Wei Hou. Heilongjiang Provincial CDC: Wei Sun, Shichun Yan, Xiaoming Cui. Nangang CDC: Chi Wang, Zhenyuan Wu, Yanjie Li, Quan Kang. Hainan Provincial CDC: Huiming Luo, Tingting Ou. Meilan CDC: Xiangyang Zheng, Zhendong Guo, Shukuan Wu, Yilei Li, Huimei Li. Jiangsu Provincial CDC: Ming Wu, Yonglin Zhou, Jinyi Zhou, Ran Tao, Jie Yang, Jian Su. Suzhou CDC: Fang Liu, Jun Zhang, Yihe Hu, Yan Lu, Liangcai Ma, Aiyu Tang, Shuo Zhang, Jianrong Jin, Jingchao Liu. Guangxi Provincial CDC: Mei Lin, Zhenzhen Lu. Liuzhou CDC: Lifang Zhou, Changping Xie, Jian Lan, Tingping Zhu, Yun Liu, Liuping Wei, Liyuan Zhou, Ningyu Chen, Yulu Qin, Sisi Wang. Sichuan Provincial CDC: Xianping Wu, Ningmei Zhang, Xiaofang Chen, Xiaoyu Chang. Pengzhou CDC: Mingqiang Yuan, Xia Wu, Xiaofang Chen, Wei Jiang, Jiaqiu Liu, Qiang Sun. Gansu Provincial CDC: Faqing Chen, Xiaolan Ren, Caixia Dong. Maiji CDC: Hui Zhang, Enke Mao, Xiaoping Wang, Tao Wang, Xi zhang. Henan Provincial CDC: Kai Kang, Shixian Feng, Huizi Tian, Lei Fan. Huixian CDC: XiaoLin Li, Huarong Sun, Pan He, Xukui Zhang. Zhejiang Provincial CDC: Min Yu, Ruying Hu, Hao Wang. Tongxiang CDC: Xiaoyi Zhang, Yuan Cao, Kaixu Xie, Lingli Chen, Dun Shen. Hunan Provincial CDC: Xiaojun Li, Donghui Jin, Li Yin, Huilin Liu, Zhongxi Fu. Liuyang CDC: Xin Xu, Hao Zhang, Jianwei Chen, Yuan Peng, Libo Zhang, Chan Qu.

Event Adjudication Clinicians:

Beijing Tiantan Hospital, Capital Medical University Shuya Li, Haiqiang Qin, Yongjun Wang, Peking University People’s Hospital Qiling Chen, Jihua Wang, The 1st Affiliated Hospital of Harbin Medical University Xiaojia Sun, Lei Wang, Xun Wang, Liming Zhang, Shanshan Zhou, The 2nd Affiliated Hospital of Harbin Medical University Hongyuan Chen, Li Chen, Haiyan Gou, Weizhi Wang, Yanmei Zhu, Yulan Zhu, The 2nd Hospital of Hebei Medical University Ning Zhang, Huashan Hospital Xin Cheng, Qiang Dong, Yi Dong, Kun Fang, Yiting Mao, Jinling Hospital Yu An, Peiling Chen, Yinghua Chen, Zhihong Liu, Lihua Zhang The People’s Hospital of Liaoning Province Xiaohong Chen, Naixin Jv, Xiaojiu Li, Liyang Liu, Yun Lu, Xiaona Xing, Qingdao Fuwai Cardiovascular Hospital Shihao You, Shengjing Hospital of China Medical University Xiaoli Cheng, Chaojun Gua, Jinping Jiang, Jingyi Liu, Shumei Ma, Shenyang Military General Hospital Xuefeng Yang, The First People’s Hospital of Shenyang Xiaomo Du, Jian Xu, Xuecheng Yang, Xiaodi Zhao, West China Hospital, Sichuan University Zilong Hao, Ming Liu, Deren Wang, The Second Affiliated Hospital of Suzhou University Xiaoting Li, Suzhou Kowloon Hospital Shanghai Jiao Tong University School of Medicine Lili Hui, Zhanling Liao, Feng Liu, Qingdao Fuwai Cardiovascular Hospital Chunning Feng, Dejiang Ji, Fengxia Qu, Wenwen Yuan, The First Affiliated Hospital of Zhengzhou University Xin Fu, Zhongshan Hospital, Jing Ding, Peng Du, Lirong Jin, Yueshi Mao, Xin Wang.
