# Supplementary material for: Adiposity and risk of breast cancer in 280 000 Chinese women: a prospective cohort study
Source: Int J Epidemiol. 2026 Aug 19;55(5):dyag155. doi: 10.1093/ije/dyag155 (PMC13489709; doi:10.1093/ije/dyag155)

Supplementary material

Adiposity and risk of breast cancer in 280,000 Chinese women: a prospective cohort study

Christiana Kartsonaki [1], Neil Wright [1], Iona Millwood [1], Yuanjie Pang [2,3,4], Dianjianyi Sun [2,3,4], Pei Pei [3], Dan Schmidt [1], Rebecca Stevens [1], Yuan Cao [5], Huaidong Du [1], Jun Lv [2,3,4], Yiping Chen [1], Canqing Yu [2,3,4], Junshi Chen [6], Derrick Bennett [1], Liming Li [2,3,4], Ling Yang* [1], Zhengming Chen* [1]

1. Clinical Trial Service Unit & Epidemiological Studies Unit (CTSU), Nuffield Department of Population Health, Big Data Institute Building, Roosevelt Drive, University of Oxford, UK
2. Department of Epidemiology and Biostatistics, School of Public Health, Peking University Health Science Center, Beijing, 100191, China
3. Peking University Center for Public Health and Epidemic Preparedness & Response, Beijing, 100191, China
4. Key Laboratory of Epidemiology of Major Diseases (Peking University), Ministry of Education
5. Tongxiang CDC, NO.64 Maodun East Road, Wutong Subdistrict, Tongxiang, Zhejiang, China
6. China National Center For Food Safety Risk Assessment, No. 37, Guangqu Road, Chaoyang District, Beijing, 100022, P.R. China

Address for correspondence:

Christiana Kartsonaki
CTSU
NDPH, Big Data Institute Building
University of Oxford
Old Road Campus
Oxford, OX3 7LF, UK
Tel: 44-1865-743626
Fax: 44-1865-743985

5 July 2026

Table of contents

Supplementary methods 4

Table S1. Tumour characteristics by age at diagnosis among 1617 validated breast cancer cases. Among 284,538 women with no history of cancer, hysterectomy, oophorectomy, or breast surgery at baseline. 5

Table S2. Incidence rates of breast cancer by age at baseline and region, per 100,000 person-years (in the full cohort). 9

Figure S1. Incidence rate (standardised by age [in 10-year age groups]) and standard error. Harbin, Qingdao, Liuzhou, Haikou, and Suzhou are urban regions. 10

Figure S2. HRs (95% CIs) for breast cancer by each adiposity measure, without and with adjustment for BMI, among women who were pre- and postmenopausal at baseline. Stratified by region and adjusted for age (numeric), smoking (never, occasional, ex regular, or current regular), alcohol drinking (never regular, ex regular, or current regular), and education (no formal/primary, middle/high school, or technical school/university). 11

Figure S3. HRs (95% CIs) for breast cancer by each adiposity measure, without and with adjustment for waist circumference, among women who were pre- and postmenopausal at baseline. Stratified by region and adjusted for age (numeric), smoking (never, occasional, ex regular, or current regular), alcohol (never regular, ex regular, or current regular), and education (no formal/primary, middle/high school, or technical school/university). 12

Figure S4. HRs (95% CIs) for breast cancer by each adiposity measure among women who were premenopausal at baseline. Stratified by region and adjusted for age (numeric), smoking (never, occasional, ex regular, or current regular), alcohol (never regular, ex regular, or current regular), education (no formal/primary, middle/high school, or technical school/university), family history of cancer (no, yes), use of oral contraceptives (never, past, current), parity (number of live births, numeric), and age at menarche (numeric). .13

Figure S5. HRs (95% CIs) for breast cancer by each adiposity measure among women who were postmenopausal at baseline. Stratified by region and adjusted for age (numeric), smoking (never, occasional, ex regular, or current regular), alcohol (never regular, ex regular, or current regular), education (no formal/primary, middle/high school, or technical school/university), family history of cancer (no, yes), use of oral contraceptives (never, past, current), parity (number of live births, numeric), and age at menarche (numeric). 14

Figure S6. HRs (95% CIs) for breast cancer by each adiposity measure, adjusting for BMI, among women who were pre- and postmenopausal at baseline, censoring premenopausal women at age 48. Models are adjusted for age (numeric), smoking status (never, occasional, ex regular, or current regular), alcohol drinking (never regular, ex regular, or current regular), education (no formal/primary, middle/high school, or technical school/university), BMI (numeric) and stratified by region. 15

Figure S7. HRs (95% CIs) for ductal and lobular breast cancer by each adiposity measure among women who were pre- and postmenopausal at baseline. Stratified by region and adjusted for adjusted for age (numeric), smoking (never, occasional, ex regular, or current regular), alcohol drinking (never regular, ex regular, or current regular), and education (no formal/primary, middle/high school, or technical school/university). 16

Figure S8. HRs (95% CIs) for (A) ER+ and (B) ER- breast cancer by BMI among women who were premenopausal at baseline. Adjusting for age (numeric), smoking status (never, occasional, ex regular, or current regular), alcohol drinking (never regular, ex regular, or current regular), education (no formal/primary, middle/high school, or technical school/university) and stratifying by region. 17

Figure S9. HRs (95% CIs) for (A) ER+ and (B) ER- breast cancer by waist circumference among women who were premenopausal at baseline. Adjusting for age (numeric), smoking status (never, occasional, ex

regular, or current regular), alcohol drinking (never regular, ex regular, or current regular), education (no formal/primary, middle/high school, or technical school/university) and stratifying by region..............................18

Figure S10. HRs (95% CIs) for HER2+ and HER2- breast cancer by each adiposity measure among women who were pre- and postmenopausal at baseline. Stratified by region and adjusted for age (numeric), smoking (never, occasional, ex regular, or current regular), alcohol drinking (never regular, ex regular, or current regular), and education (no formal/primary, middle/high school, or technical school/university).........................19

Figure S11. HRs (95% CIs) for breast cancer by BMI (per 5 kg/m^2) in subgroups. Adjusting for age (numeric) and menopause status (pre-, peri-, or postmenopausal) and stratifying by region. 20

Figure S12. HRs (95% CIs) for breast cancer by waist circumference (per 10 cm) in subgroups. Adjusting for age (numeric) and menopause status (pre-, peri-, or postmenopausal) and stratifying by region..................21

Supplementary methods

Event validation

Additional information on cancer clinical diagnosis has been collected as part of an ongoing cancer validation programme, which involves retrieving original medical records from hospitals for any reported cancer cases to confirm the cancer diagnosis along with further detailed clinical cancer diagnosis information collection, e.g. cancer stage, grade, ER, PR and HER2 status, and histopathological subtype. By 1.1.2018, among the full cohort 1,945 cases of breast cancer had been validated (that is, also identified as breast cancer cases by the cancer validation programme), of which 1281 had some subtype information available. Stage was inferred from tumour, nodes, metastasis (TNM) stage based on AJCC 8th edition (Amin et al., 2017). ER, PR, and HER2 status were recorded as strongly positive, positive, borderline, negative, or unknown. Cancers were considered to be ER positive (ER+) or PR positive (PR+) if they were strongly positive, positive, or borderline, and ER negative (ER-) or PR negative (PR-) if reported as negative. Cancers were considered to be HER2 positive (HER2+) if they were strongly positive and HER2 negative (HER2-) if they were weakly positive, borderline or negative (Wolff et al., 2013).

Statistical analysis

We excluded women with oophorectomy as we did not have information on whether the oophorectomy was unilateral or bilateral, and bilateral oophorectomy would be expected to substantially affect risk of breast cancer, as well as adiposity. We excluded women with hysterectomy because if the hysterectomy had been done before menopause and without a bilateral oophorectomy, the menopause status of participants would not have been known. Additionally, the indication for the hysterectomy is not known, therefore we excluded women with either hysterectomy or oophorectomy to reduce confounding.

References:

Amin, M.B., Edge, S., Greene, F., Byrd, D.R., Brookland, R.K., Washington, M.K., Gershenwald, J.E., Compton, C.C., Hess, K.R., Sullivan, D.C., Jessup, J.M., Brierley, J.D., Gaspar, L.E., Schilsky, R.L., Balch, C.M., Winchester, D.P., Asare, E.A., Madera, M., Gress, D.M., Meyer, L.R. (Eds.), 2017. AJCC Cancer Staging Manual, 8th ed. Springer International Publishing.

Wolff, A.C., Hammond, M.E.H., Hicks, D.G., Dowsett, M., McShane, L.M., Allison, K.H., Allred, D.C., Bartlett, J.M.S., Bilous, M., Fitzgibbons, P., Hanna, W., Jenkins, R.B., Mangu, P.B., Paik, S., Perez, E.A., Press, M.F., Spears, P.A., Vance, G.H., Viale, G., Hayes, D.F., American Society of Clinical Oncology, College of American Pathologists, 2013. Recommendations for human epidermal growth factor receptor 2 testing in breast cancer: American Society of Clinical Oncology/College of American Pathologists clinical practice guideline update. J. Clin. Oncol. Off. J. Am. Soc. Clin. Oncol. 31, 3997–4013. https://doi.org/10.1200/JCO.2013.50.9984

Table S1. Tumour characteristics by age at diagnosis among 1617 validated breast cancer cases. Among 284,538 women with no history of cancer, hysterectomy, oophorectomy, or breast surgery at baseline.

		**Age at diagnosis (years)**				
		**30-44**	**45-54**	**55-69**	**≥70**	**Total**
**Total N (%)**		151 (9.3)	560 (34.6)	718 (44.4)	188 (11.6)	1617
**ER status**	**Strongly positive**	15 (9.9)	60 (10.7)	105 (14.6)	24 (12.8)	204 (12.6)
	**Weakly positive**	24 (15.9)	99 (17.7)	160 (22.3)	44 (23.4)	327 (20.2)
	**Borderline**	1 (0.7)	3 (0.5)	4 (0.6)	5 (2.7)	13 (0.8)
	**Negative**	10 (6.6)	90 (16.1)	112 (15.6)	18 (9.6)	230 (14.2)
	(Missing)	101 (66.9)	308 (55.0)	337 (46.9)	97 (51.6)	843 (52.1)
**PR status**	**Strongly positive**	12 (7.9)	45 (8.0)	67 (9.3)	21 (11.2)	145 (9.0)
	**Weakly positive**	25 (16.6)	92 (16.4)	152 (21.2)	42 (22.3)	311 (19.2)
	**Borderline**	1 (0.7)	2 (0.4)	9 (1.3)	6 (3.2)	18 (1.1)
	**Negative**	11 (7.3)	113 (20.2)	154 (21.4)	21 (11.2)	299 (18.5)
	(Missing)	102 (67.5)	308 (55.0)	336 (46.8)	98 (52.1)	844 (52.2)
**HER2 status**	**Strongly positive**	20 (13.2)	97 (17.3)	129 (18.0)	24 (12.8)	270 (16.7)
	**Weakly positive**	7 (4.6)	52 (9.3)	67 (9.3)	25 (13.3)	151 (9.3)
	**Borderline**	0 (0.0)	5 (0.9)	5 (0.7)	2 (1.1)	12 (0.7)
	**Negative**	12 (7.9)	45 (8.0)	88 (12.3)	22 (11.7)	167 (10.3)
	(Missing)	112 (74.2)	361 (64.5)	429 (59.7)	115 (61.2)	1017 (62.9)
**Grade**	**1 (Well differentiated)**	1 (0.7)	4 (0.7)	4 (0.6)	1 (0.5)	10 (0.6)
	**2 (Moderately differentiated)**	3 (2.0)	19 (3.4)	23 (3.2)	6 (3.2)	51 (3.2)
	**3 (Poorly differentiated)**	1 (0.7)	17 (3.0)	14 (1.9)	3 (1.6)	35 (2.2)
	(Missing)	146 (96.7)	520 (92.9)	677 (94.3)	178 (94.7)	1521 (94.1)
**Histology**	**Ductal carcinoma**	48 (31.8)	234 (41.8)	342 (47.6)	75 (39.9)	699 (43.2)
	**Lobular carcinoma**	3 (2.0)	15 (2.7)	14 (1.9)	3 (1.6)	35 (2.2)
	**Other**	6 (4.0)	62 (11.1)	84 (11.7)	27 (14.4)	179 (11.1)
	**Unknown**	8 (5.3)	9 (1.6)	23 (3.2)	7 (3.7)	47 (2.9)
	(Missing)	86 (57.0)	240 (42.9)	255 (35.5)	76 (40.4)	657 (40.6)
**Sub-site**	**Axillary tail**	0 (0.0)	2 (0.4)	3 (0.4)	0 (0.0)	5 (0.3)
	**Breast**	58 (38.4)	273 (48.8)	368 (51.3)	117 (62.2)	816 (50.5)
	**Central portion**	0 (0.0)	6 (1.1)	4 (0.6)	0 (0.0)	10 (0.6)
	**Lower-inner quadrant**	0 (0.0)	4 (0.7)	11 (1.5)	4 (2.1)	19 (1.2)
	**Lower-outer quadrant**	3 (2.0)	5 (0.9)	9 (1.3)	0 (0.0)	17 (1.1)
	**Nipple**	0 (0.0)	6 (1.1)	12 (1.7)	0 (0.0)	18 (1.1)

		Age at diagnosis (years)				
		**30-44**	**45-54**	**55-69**	**≥70**	**Total**
	**Non-breast**	0 (0.0)	3 (0.5)	4 (0.6)	2 (1.1)	9 (0.6)
	**Overlapping lesion**	1 (0.7)	3 (0.5)	11 (1.5)	2 (1.1)	17 (1.1)
	**Upper-inner quadrant**	6 (4.0)	15 (2.7)	22 (3.1)	6 (3.2)	49 (3.0)
	**Upper-outer quadrant**	6 (4.0)	50 (8.9)	61 (8.5)	9 (4.8)	126 (7.8)
	(Missing)	77 (51.0)	193 (34.5)	213 (29.7)	48 (25.5)	531 (32.8)
**T stage**	**0**	0 (0.0)	2 (0.4)	3 (0.4)	0 (0.0)	5 (0.3)
	**1**	3 (2.0)	27 (4.8)	51 (7.1)	5 (2.7)	86 (5.3)
	**2**	10 (6.6)	57 (10.2)	86 (12.0)	14 (7.4)	167 (10.3)
	**3**	0 (0.0)	6 (1.1)	11 (1.5)	1 (0.5)	18 (1.1)
	**4**	0 (0.0)	1 (0.2)	11 (1.5)	1 (0.5)	13 (0.8)
	(Missing)	138 (91.4)	467 (83.4)	556 (77.4)	167 (88.8)	1328 (82.1)
**N stage**	**0**	6 (4.0)	60 (10.7)	91 (12.7)	11 (5.9)	168 (10.4)
	**1**	4 (2.6)	22 (3.9)	40 (5.6)	5 (2.7)	71 (4.4)
	**2**	2 (1.3)	11 (2.0)	19 (2.6)	2 (1.1)	34 (2.1)
	**3**	1 (0.7)	5 (0.9)	15 (2.1)	1 (0.5)	22 (1.4)
	(Missing)	138 (91.4)	462 (82.5)	553 (77.0)	169 (89.9)	1322 (81.8)
**M stage**	**0**	13 (8.6)	95 (17.0)	154 (21.4)	19 (10.1)	281 (17.4)
	**1**	0 (0.0)	0 (0.0)	8 (1.1)	0 (0.0)	8 (0.5)
	(Missing)	138 (91.4)	465 (83.0)	556 (77.4)	169 (89.9)	1328 (82.1)
**TNM stage**	**T0 N0 M0**	0 (0.0)	1 (0.2)	2 (0.3)	0 (0.0)	3 (0.2)
	**T0 N1 M0**	0 (0.0)	1 (0.2)	1 (0.1)	0 (0.0)	2 (0.1)
	**T1 N0 M0**	3 (2.0)	20 (3.6)	39 (5.4)	4 (2.1)	66 (4.1)
	**T1 N0 Mx**	0 (0.0)	1 (0.2)	0 (0.0)	0 (0.0)	1 (0.1)
	**T1 N1 M0**	0 (0.0)	1 (0.2)	6 (0.8)	1 (0.5)	8 (0.5)
	**T1 N2 M0**	0 (0.0)	2 (0.4)	3 (0.4)	0 (0.0)	5 (0.3)
	**T1 N2 Mx**	0 (0.0)	1 (0.2)	0 (0.0)	0 (0.0)	1 (0.1)
	**T1 N3 M0**	0 (0.0)	1 (0.2)	2 (0.3)	0 (0.0)	3 (0.2)
	**T1 N3 Mx**	0 (0.0)	1 (0.2)	1 (0.1)	0 (0.0)	2 (0.1)
	**T2 N0 M0**	3 (2.0)	31 (5.5)	42 (5.8)	5 (2.7)	81 (5.0)
	**T2 N0 Mx**	0 (0.0)	0 (0.0)	1 (0.1)	1 (0.5)	2 (0.1)
	**T2 N1 M0**	4 (2.6)	19 (3.4)	21 (2.9)	3 (1.6)	47 (2.9)
	**T2 N1 M1**	0 (0.0)	0 (0.0)	2 (0.3)	0 (0.0)	2 (0.1)
	**T2 N1 Mx**	0 (0.0)	0 (0.0)	1 (0.1)	0 (0.0)	1 (0.1)

		Age at diagnosis (years)				
		**30-44**	**45-54**	**55-69**	**≥70**	**Total**
	**T2 N2 M0**	2 (1.3)	7 (1.2)	13 (1.8)	2 (1.1)	24 (1.5)
	**T2 N3 M0**	1 (0.7)	0 (0.0)	4 (0.6)	1 (0.5)	6 (0.4)
	**T2 N3 M1**	0 (0.0)	0 (0.0)	2 (0.3)	0 (0.0)	2 (0.1)
	**T2 Nx M0**	0 (0.0)	0 (0.0)	0 (0.0)	1 (0.5)	1 (0.1)
	**T2 Nx Mx**	0 (0.0)	0 (0.0)	0 (0.0)	1 (0.5)	1 (0.1)
	**T3 N0 M0**	0 (0.0)	3 (0.5)	2 (0.3)	0 (0.0)	5 (0.3)
	**T3 N1 M0**	0 (0.0)	0 (0.0)	4 (0.6)	1 (0.5)	5 (0.3)
	**T3 N1 Mx**	0 (0.0)	0 (0.0)	1 (0.1)	0 (0.0)	1 (0.1)
	**T3 N2 M0**	0 (0.0)	1 (0.2)	1 (0.1)	0 (0.0)	2 (0.1)
	**T3 N3 M0**	0 (0.0)	0 (0.0)	2 (0.3)	0 (0.0)	2 (0.1)
	**T3 N3 M1**	0 (0.0)	0 (0.0)	1 (0.1)	0 (0.0)	1 (0.1)
	**T3 N3 Mx**	0 (0.0)	1 (0.2)	0 (0.0)	0 (0.0)	1 (0.1)
	**T3 Nx M0**	0 (0.0)	1 (0.2)	0 (0.0)	0 (0.0)	1 (0.1)
	**T4 N0 M0**	0 (0.0)	0 (0.0)	2 (0.3)	1 (0.5)	3 (0.2)
	**T4 N1 M0**	0 (0.0)	0 (0.0)	3 (0.4)	0 (0.0)	3 (0.2)
	**T4 N1 M1**	0 (0.0)	0 (0.0)	1 (0.1)	0 (0.0)	1 (0.1)
	**T4 N2 M0**	0 (0.0)	0 (0.0)	2 (0.3)	0 (0.0)	2 (0.1)
	**T4 N3 M0**	0 (0.0)	1 (0.2)	2 (0.3)	0 (0.0)	3 (0.2)
	**T4 Nx M1**	0 (0.0)	0 (0.0)	1 (0.1)	0 (0.0)	1 (0.1)
	**Tx N0 M0**	0 (0.0)	4 (0.7)	3 (0.4)	0 (0.0)	7 (0.4)
	**Tx N1 M0**	0 (0.0)	1 (0.2)	0 (0.0)	0 (0.0)	1 (0.1)
	**Tx N3 M0**	0 (0.0)	1 (0.2)	0 (0.0)	0 (0.0)	1 (0.1)
	**Tx N3 M1**	0 (0.0)	0 (0.0)	1 (0.1)	0 (0.0)	1 (0.1)
	(Missing)	138 (91.4)	461 (82.3)	552 (76.9)	167 (88.8)	1318 (81.5)
**Numeric**	**1A**	3 (2.0)	20 (3.6)	39 (5.4)	4 (2.1)	66 (4.1)
**stage**	**2A**	3 (2.0)	33 (5.9)	49 (6.8)	6 (3.2)	91 (5.6)
	**2B**	4 (2.6)	22 (3.9)	23 (3.2)	3 (1.6)	52 (3.2)
	**3A**	2 (1.3)	10 (1.8)	21 (2.9)	3 (1.6)	36 (2.2)
	**3B**	0 (0.0)	0 (0.0)	7 (1.0)	1 (0.5)	8 (0.5)
	**3C**	1 (0.7)	3 (0.5)	10 (1.4)	1 (0.5)	15 (0.9)
	**4**	0 (0.0)	0 (0.0)	8 (1.1)	0 (0.0)	8 (0.5)
	(Missing)	138 (91.4)	472 (84.3)	561 (78.1)	170 (90.4)	1341 (82.9)
**Numeric**	**1**	3 (2.0)	20 (3.6)	39 (5.4)	4 (2.1)	66 (4.1)

		Age at diagnosis (years)				
		**30-44**	**45-54**	**55-69**	**≥70**	**Total**
**stage**	**2**	7 (4.6)	55 (9.8)	72 (10.0)	9 (4.8)	143 (8.8)
**(simplified)**	**3**	3 (2.0)	13 (2.3)	38 (5.3)	5 (2.7)	59 (3.6)
	**4**	0 (0.0)	0 (0.0)	8 (1.1)	0 (0.0)	8 (0.5)
	(Missing)	138 (91.4)	472 (84.3)	561 (78.1)	170 (90.4)	1341 (82.9)
**ER status**	**-**	10 (6.6)	90 (16.1)	112 (15.6)	18 (9.6)	230 (14.2)
	**+**	40 (26.5)	162 (28.9)	269 (37.5)	73 (38.8)	544 (33.6)
	(Missing)	101 (66.9)	308 (55.0)	337 (46.9)	97 (51.6)	843 (52.1)
**PR status**	**-**	11 (7.3)	113 (20.2)	154 (21.4)	21 (11.2)	299 (18.5)
	**+**	38 (25.2)	139 (24.8)	228 (31.8)	69 (36.7)	474 (29.3)
	(Missing)	102 (67.5)	308 (55.0)	336 (46.8)	98 (52.1)	844 (52.2)
**HER2 status**	**-**	19 (12.6)	102 (18.2)	160 (22.3)	49 (26.1)	330 (20.4)
	**+**	20 (13.2)	97 (17.3)	129 (18.0)	24 (12.8)	270 (16.7)
	(Missing)	112 (74.2)	361 (64.5)	429 (59.7)	115 (61.2)	1017 (62.9)
**ER/PR/HER2**	**ER- PR- HER2-**	4 (2.6)	30 (5.4)	33 (4.6)	6 (3.2)	73 (4.5)
	**ER- PR- HER2+**	4 (2.6)	31 (5.5)	44 (6.1)	4 (2.1)	83 (5.1)
	**ER- PR+ HER2-**	1 (0.7)	4 (0.7)	6 (0.8)	0 (0.0)	11 (0.7)
	**ER- PR+ HER2+**	0 (0.0)	2 (0.4)	2 (0.3)	0 (0.0)	4 (0.2)
	**ER+ PR- HER2-**	0 (0.0)	11 (2.0)	21 (2.9)	1 (0.5)	33 (2.0)
	**ER+ PR- HER2+**	1 (0.7)	16 (2.9)	22 (3.1)	1 (0.5)	40 (2.5)
	**ER+ PR+ HER2-**	14 (9.3)	56 (10.0)	98 (13.6)	40 (21.3)	208 (12.9)
	**ER+ PR+ HER2+**	15 (9.9)	48 (8.6)	61 (8.5)	19 (10.1)	143 (8.8)
	(Missing)	112 (74.2)	362 (64.6)	431 (60.0)	117 (62.2)	1022 (63.2)

Table S2. Incidence rates of breast cancer by age at baseline and region, per 100,000 person-years (in the full cohort).

	Number of breast cancer cases	Person-years	Crude incidence rate	Standardised incidence rate	Standard error of standardised incidence rate
**Overall**	3,108	3,583,905.8	86.7	86.7	1.6
**Age**					
30-39	393	589,743.9	66.6	76.4	4.0
40-49	1,049	1,134,645.3	92.5	92.7	2.9
50-59	1,074	1,119,137.4	96.0	96.5	3.0
60-69	455	569,495.7	79.9	73.8	3.5
70-79	137	170,883.4	80.2	65.4	5.9
**Region**					
Qingdao	364	234,555.7	155.2	154.9	8.2
Harbin	685	401,509.7	170.6	170.2	6.6
Haikou	235	216,850.2	108.4	112.1	7.4
Suzhou	274	370,305.7	74.0	74.1	4.5
Liuzhou	500	357,342.3	139.9	139.2	6.4
Sichuan	169	406,524.0	41.6	41.5	3.2
Gansu	78	362,165.0	21.5	22.5	2.6
Henan	274	421,559.7	65.0	64.5	3.9
Zhejiang	329	413,208.9	79.6	80.4	4.5
Hunan	200	399,884.7	50.0	49.6	3.5

Incidence rates by age are standardised by region, and incidence rates by region are standardised by age.

Figure S1. Incidence rate (standardised by age [in 10-year age groups]) and standard error. Harbin, Qingdao, Liuzhou, Haikou, and Suzhou are urban regions.

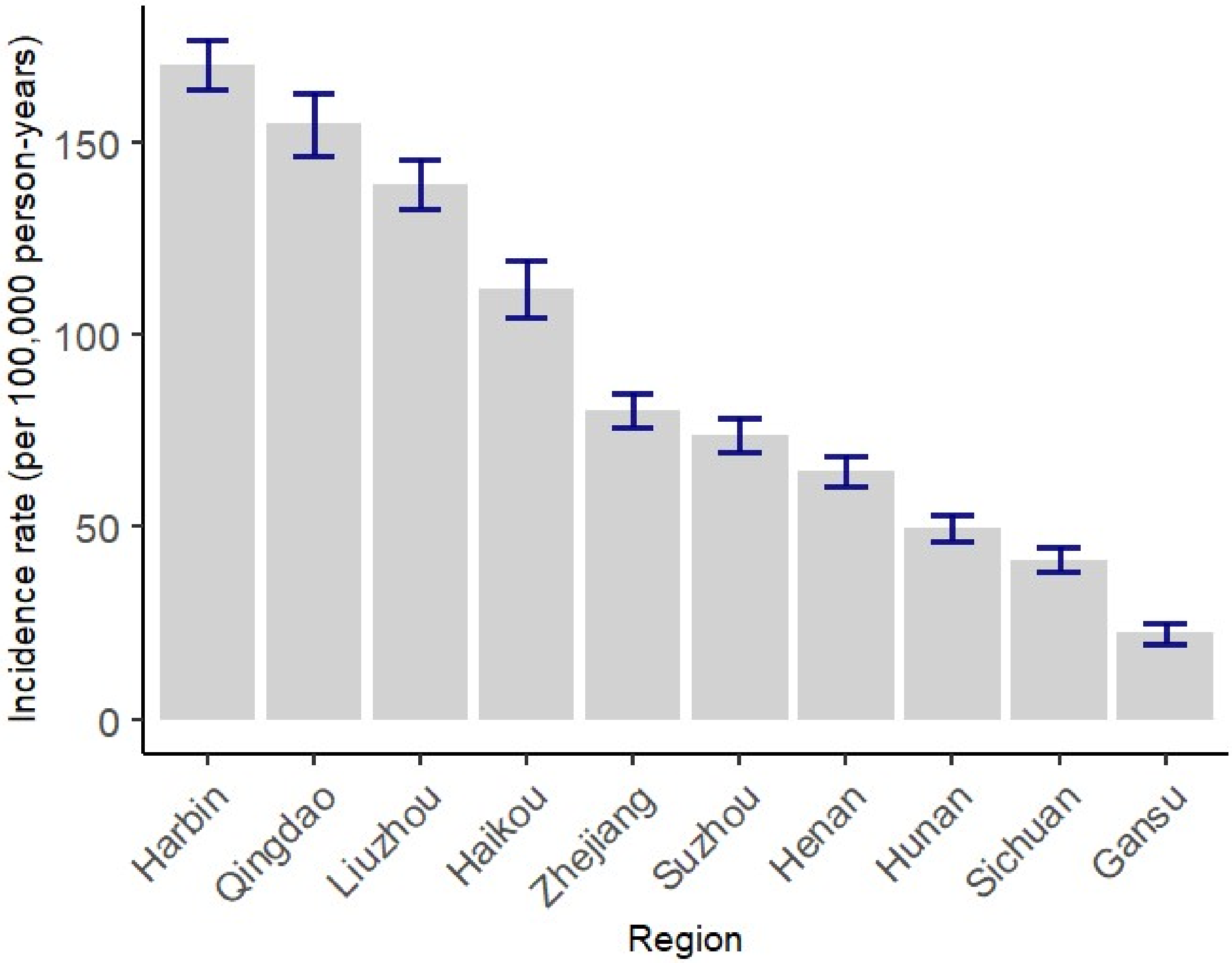

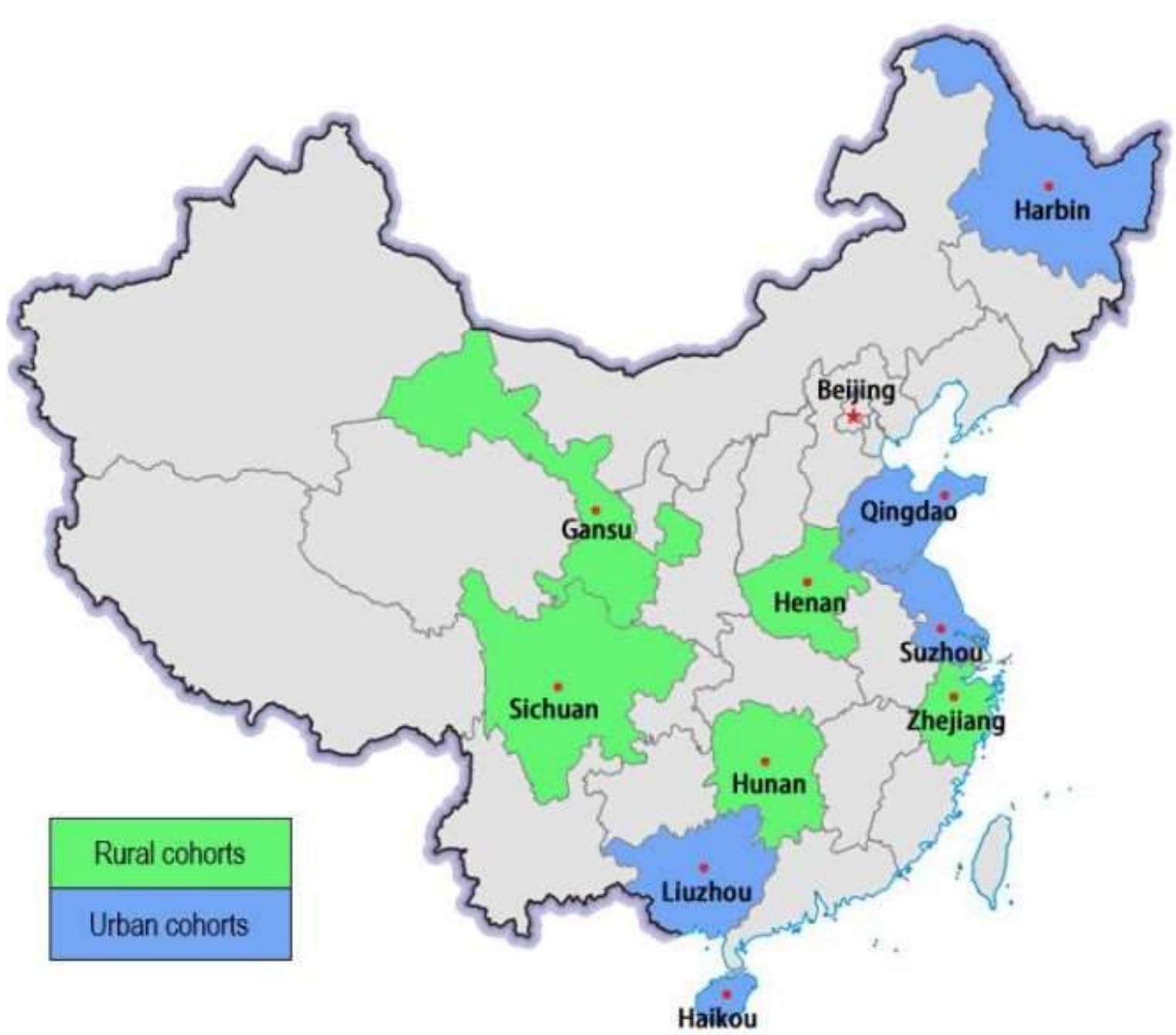

Figure S2. HRs (95% CIs) for breast cancer by each adiposity measure, without and with adjustment for BMI, among women who were pre- and postmenopausal at baseline. Stratified by region and adjusted for age (numeric), smoking (never, occasional, ex regular, or current regular), alcohol drinking (never regular, ex regular, or current regular), and education (no formal/primary, middle/high school, or technical school/university).

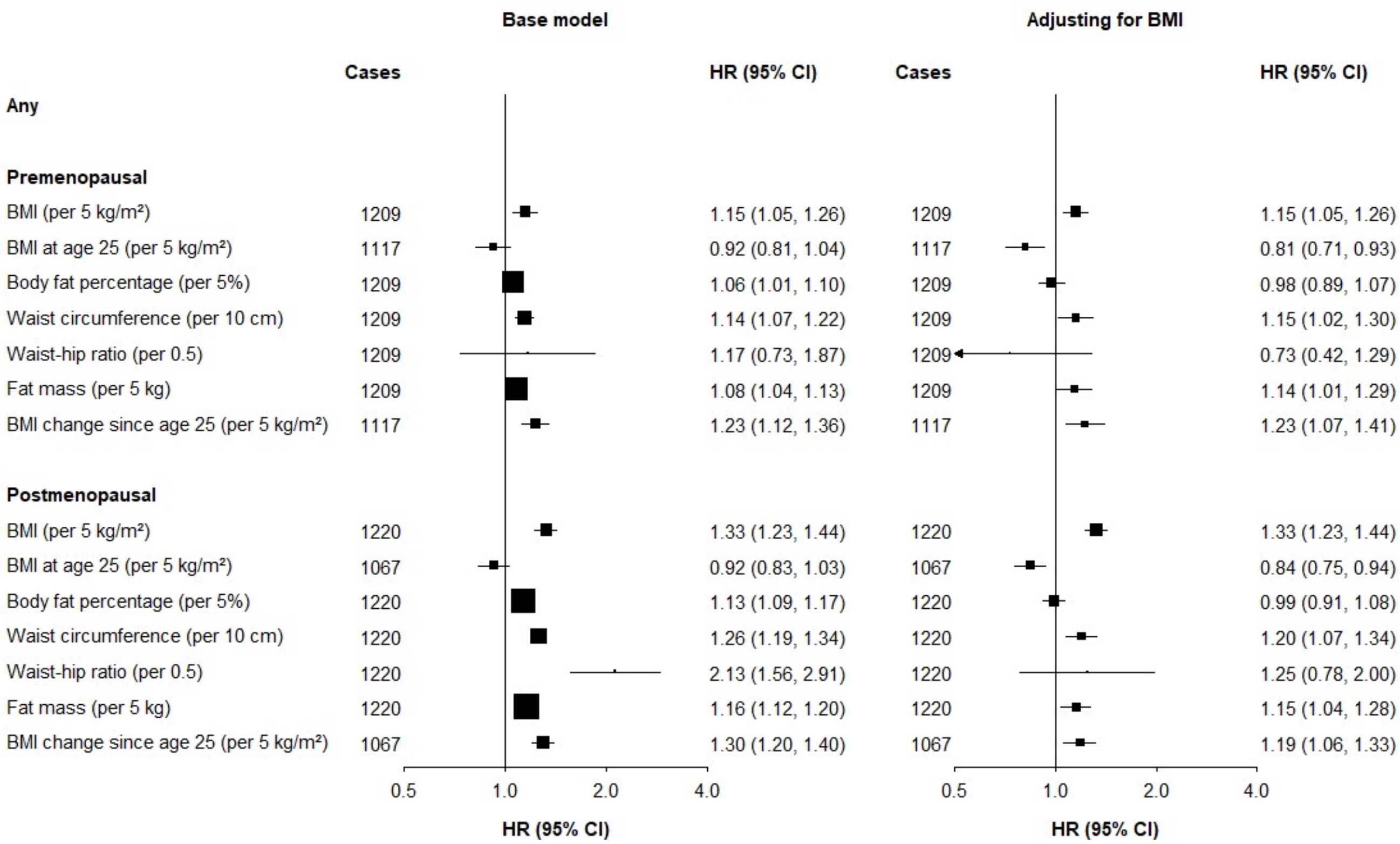

Figure S3. HRs (95% CIs) for breast cancer by each adiposity measure, without and with adjustment for waist circumference, among women who were pre- and postmenopausal at baseline. Stratified by region and adjusted for age (numeric), smoking (never, occasional, ex regular, or current regular), alcohol (never regular, ex regular, or current regular), and education (no formal/primary, middle/high school, or technical school/university).

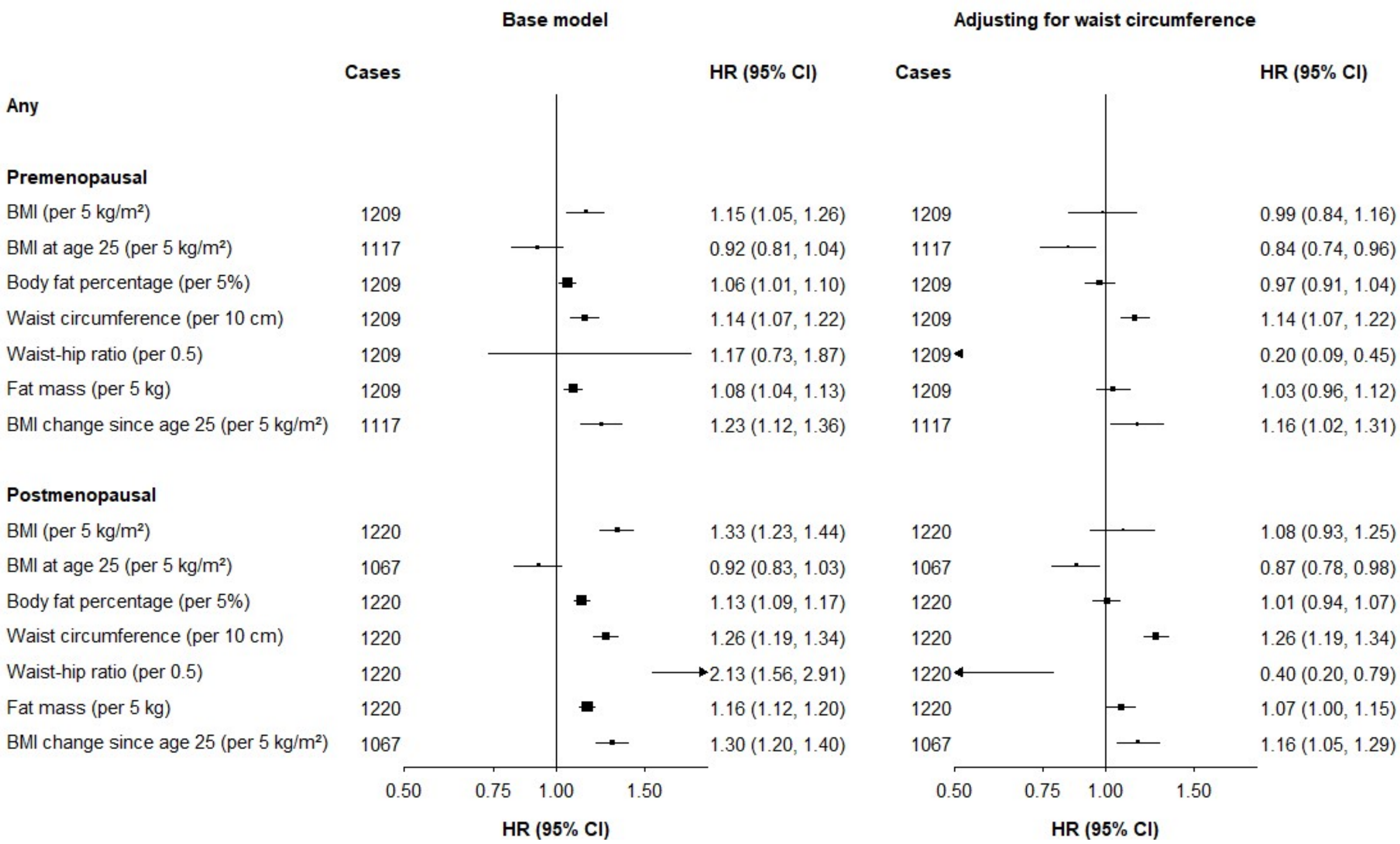

Figure S4. HRs (95% CIs) for breast cancer by each adiposity measure among women who were premenopausal at baseline. Stratified by region and adjusted for age (numeric), smoking (never, occasional, ex regular, or current regular), alcohol (never regular, ex regular, or current regular), education (no formal/primary, middle/high school, or technical school/university), family history of cancer (no, yes), use of oral contraceptives (never, past, current), parity (number of live births, numeric), and age at menarche (numeric).

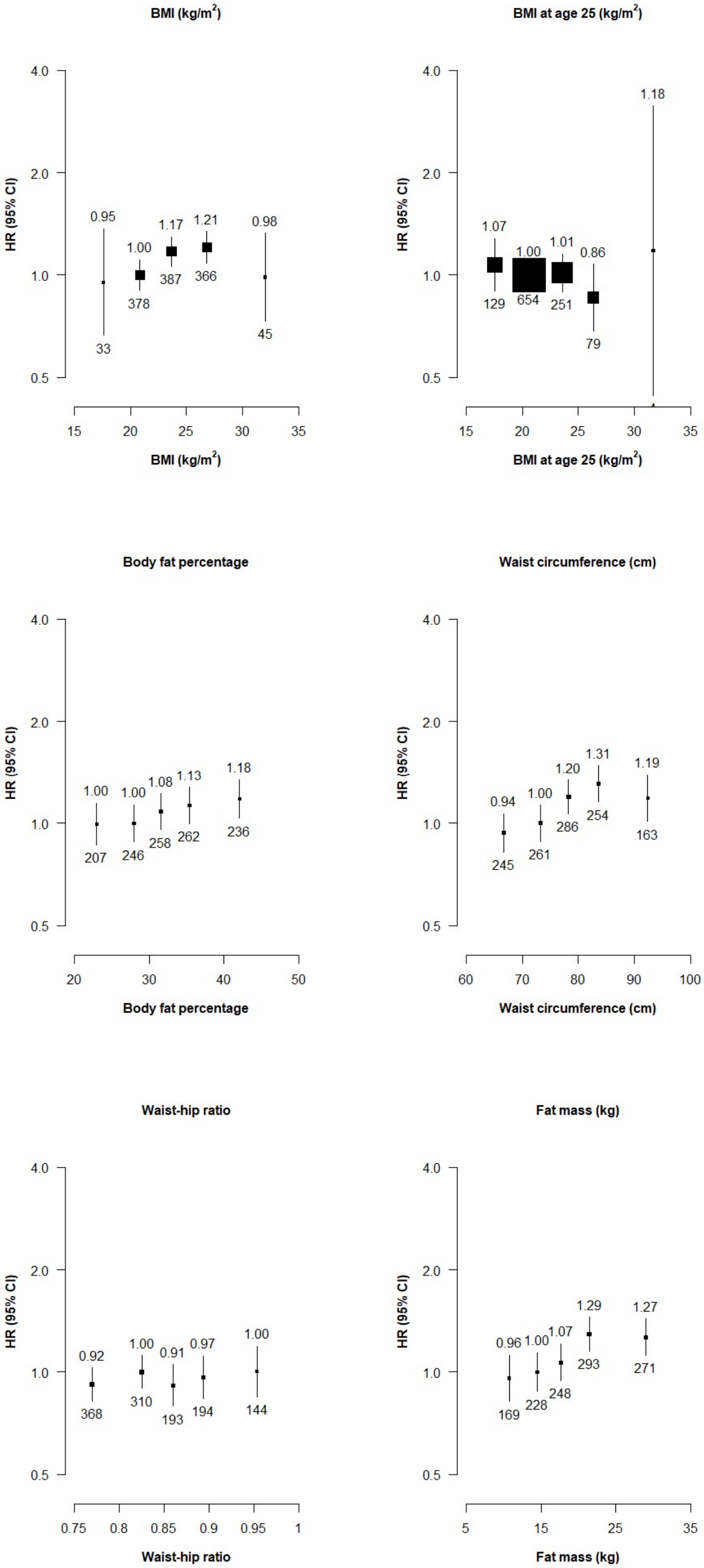

Figure S5. HRs (95% CIs) for breast cancer by each adiposity measure among women who were postmenopausal at baseline. Stratified by region and adjusted for age (numeric), smoking (never, occasional, ex regular, or current regular), alcohol (never regular, ex regular, or current regular), education (no formal/primary, middle/high school, or technical school/university), family history of cancer (no, yes), use of oral

, numeric), and age at menarche (numeric).

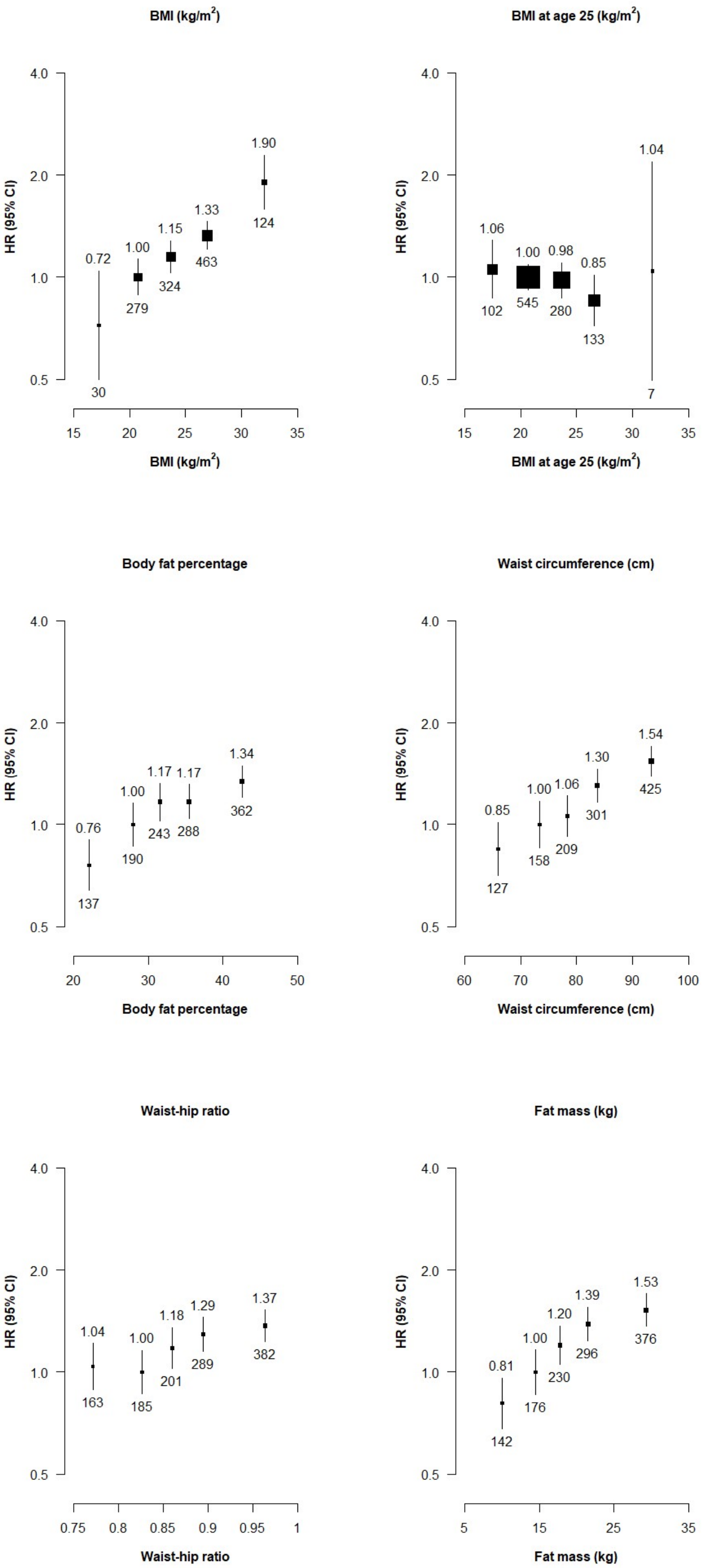

Figure S6. HRs (95% CIs) for breast cancer by each adiposity measure, adjusting for BMI, among women who were pre- and postmenopausal at baseline, censoring premenopausal women at age 48. Models are adjusted for age (numeric), smoking status (never, occasional, ex regular, or current regular), alcohol drinking (never regular, ex regular, or current regular), education (no formal/primary, middle/high school, or technical school/university), BMI (numeric) and stratified by region.

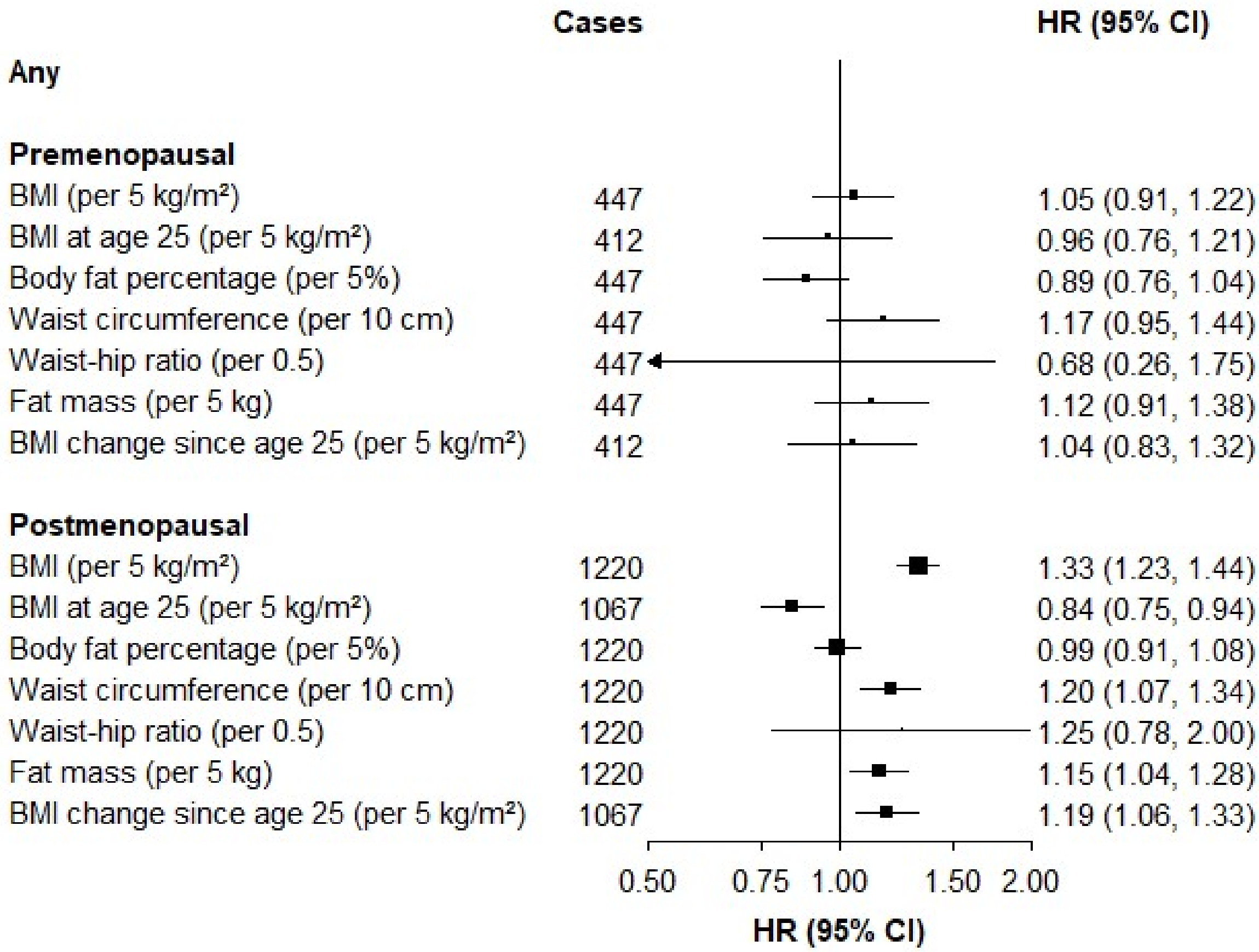

Figure S7. HRs (95% CIs) for ductal and lobular breast cancer by each adiposity measure among women who were pre- and postmenopausal at baseline. Stratified by region and adjusted for adjusted for age (numeric), smoking (never, occasional, ex regular, or current regular), alcohol drinking (never regular, ex regular, or current regular), and education (no formal/primary, middle/high school, or technical school/university).

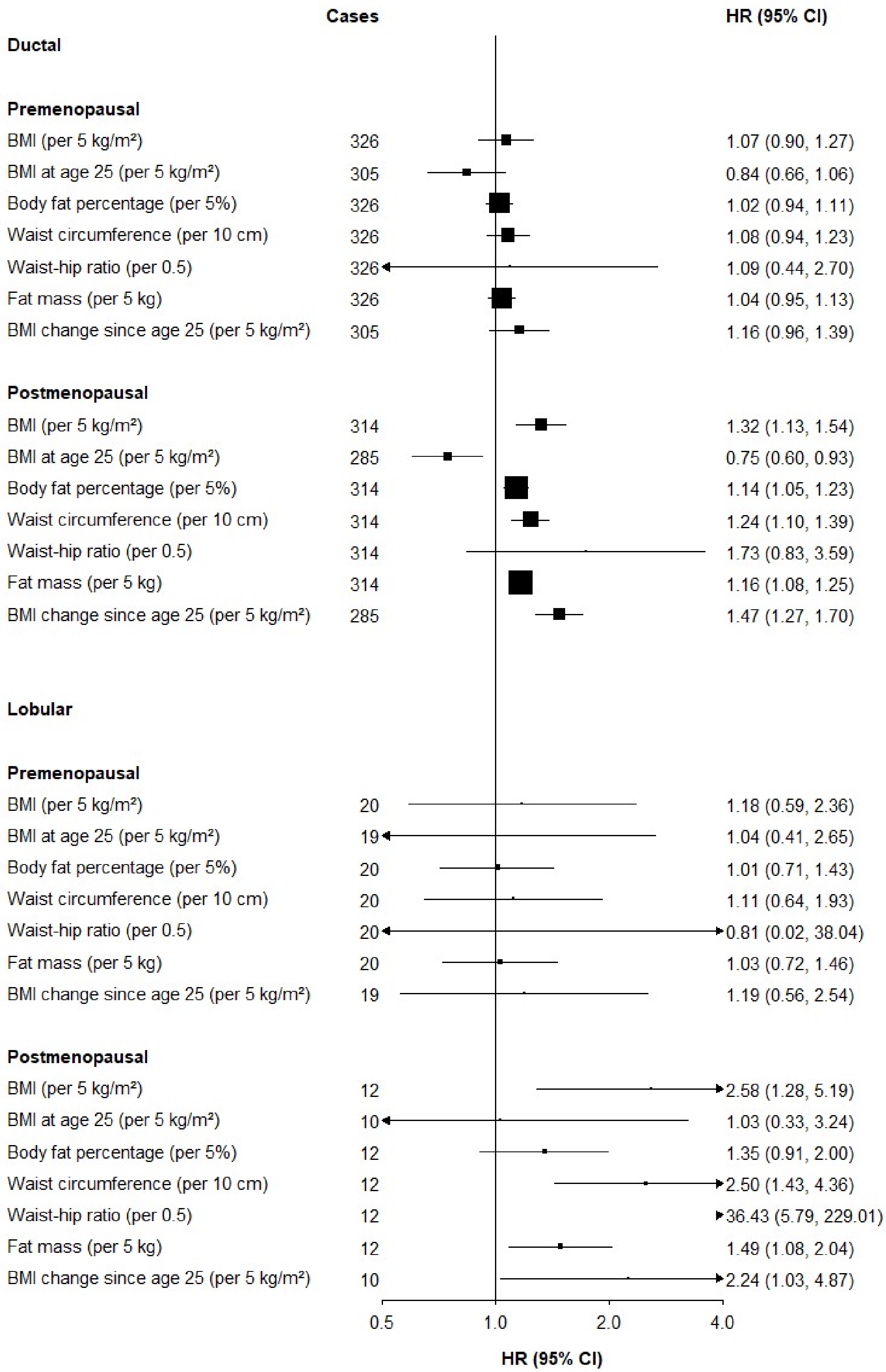

Figure S8. HRs (95% CIs) for (A) ER+ and (B) ER- breast cancer by BMI among women who were premenopausal at baseline. Adjusting for age (numeric), smoking status (never, occasional, ex regular, or current regular), alcohol drinking (never regular, ex regular, or current regular), education (no formal/primary, middle/high school, or technical school/university) and stratifying by region.

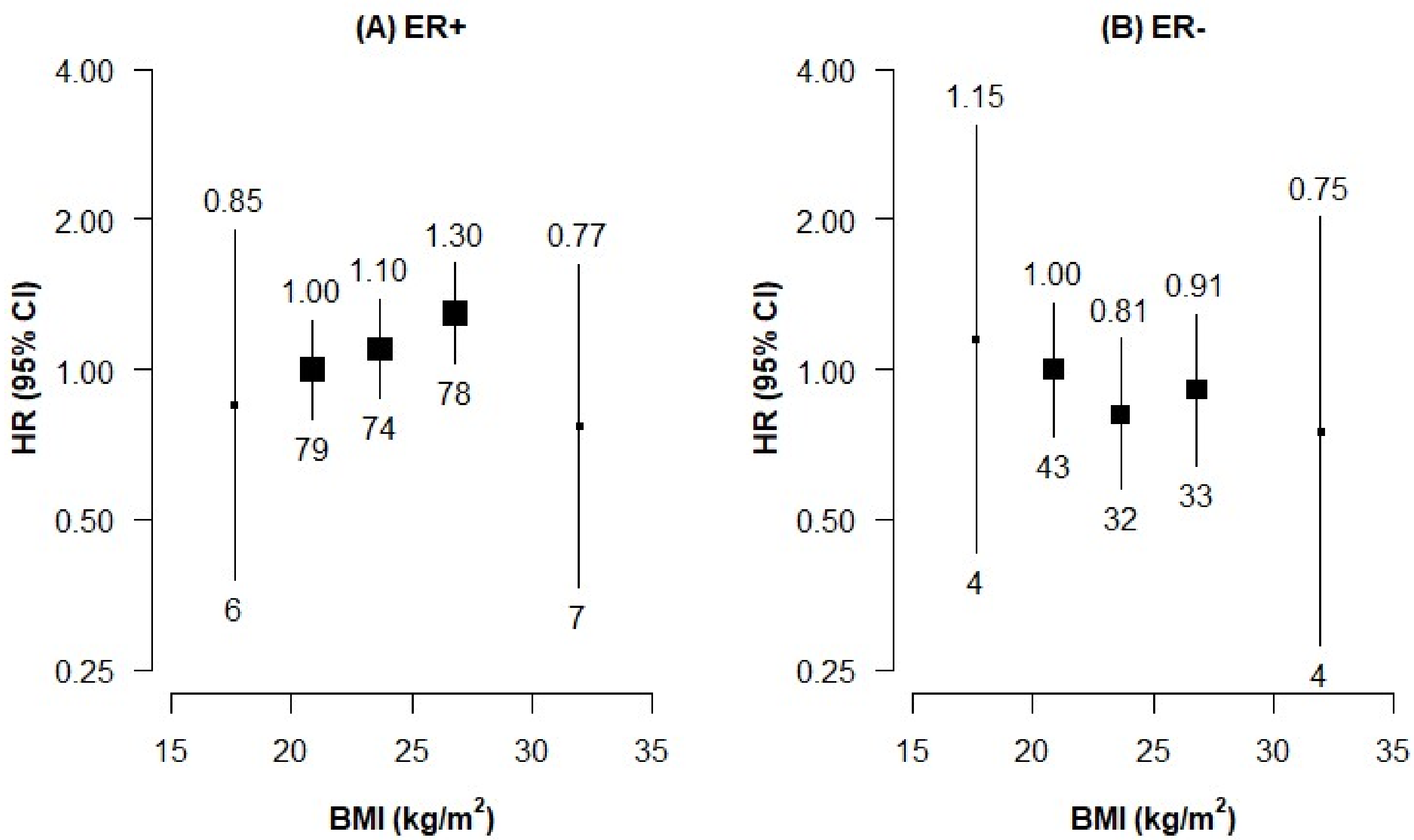

Figure S9. HRs (95% CIs) for (A) ER+ and (B) ER- breast cancer by waist circumference among women who were premenopausal at baseline. Adjusting for age (numeric), smoking status (never, occasional, ex regular, or current regular), alcohol drinking (never regular, ex regular, or current regular), education (no formal/primary, middle/high school, or technical school/university) and stratifying by region.

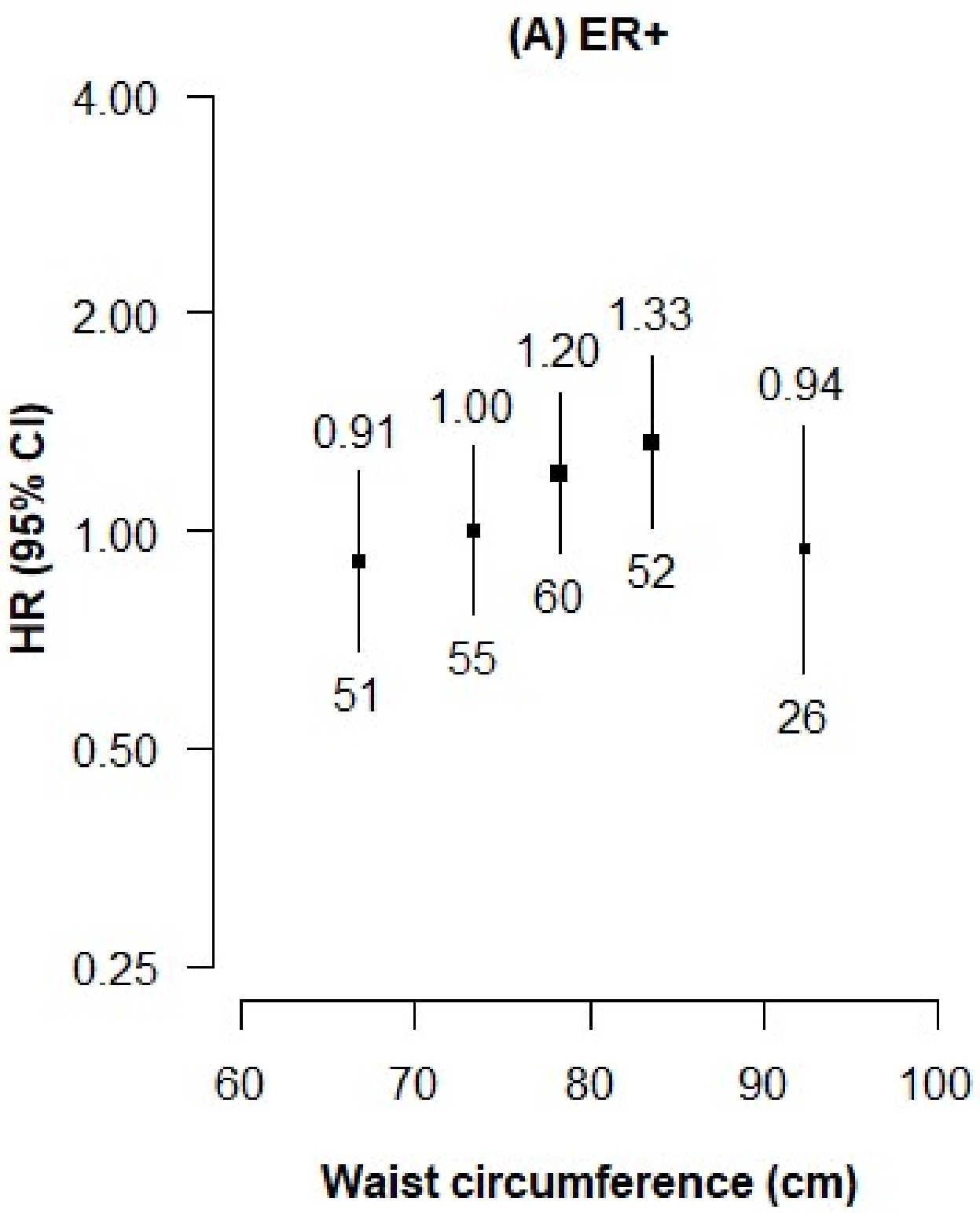

Figure S10. HRs (95% CIs) for HER2+ and HER2- breast cancer by each adiposity measure among women who were pre- and postmenopausal at baseline. Stratified by region and adjusted for age (numeric), smoking (never, occasional, ex regular, or current regular), alcohol drinking (never regular, ex regular, or current regular), and education (no formal/primary, middle/high school, or technical school/university).

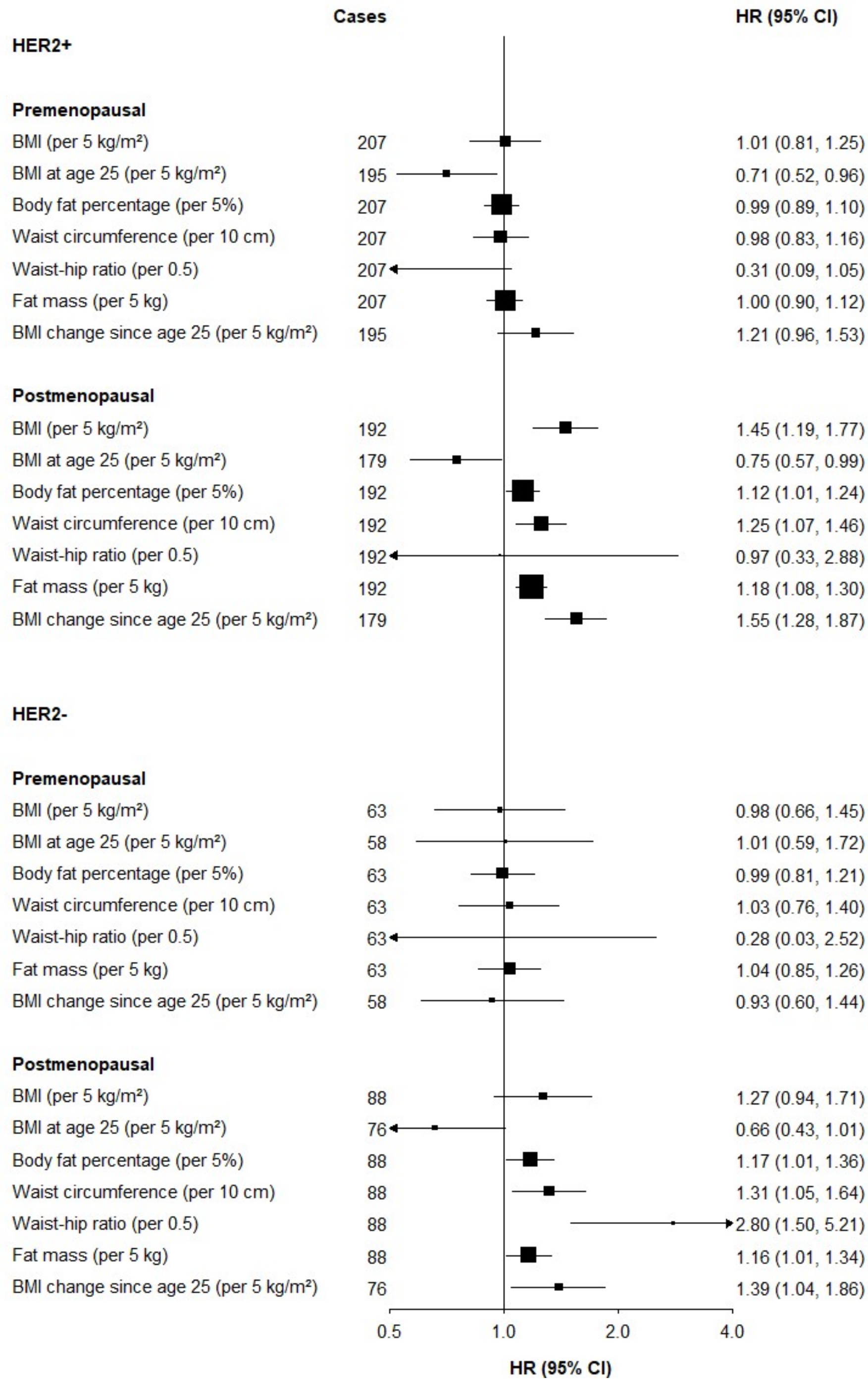

Figure S11. HRs (95% CIs) for breast cancer by BMI (per 5 kg/m^2) in subgroups. Adjusting for age (numeric) and menopause status (pre-, peri-, or postmenopausal) and stratifying by region.

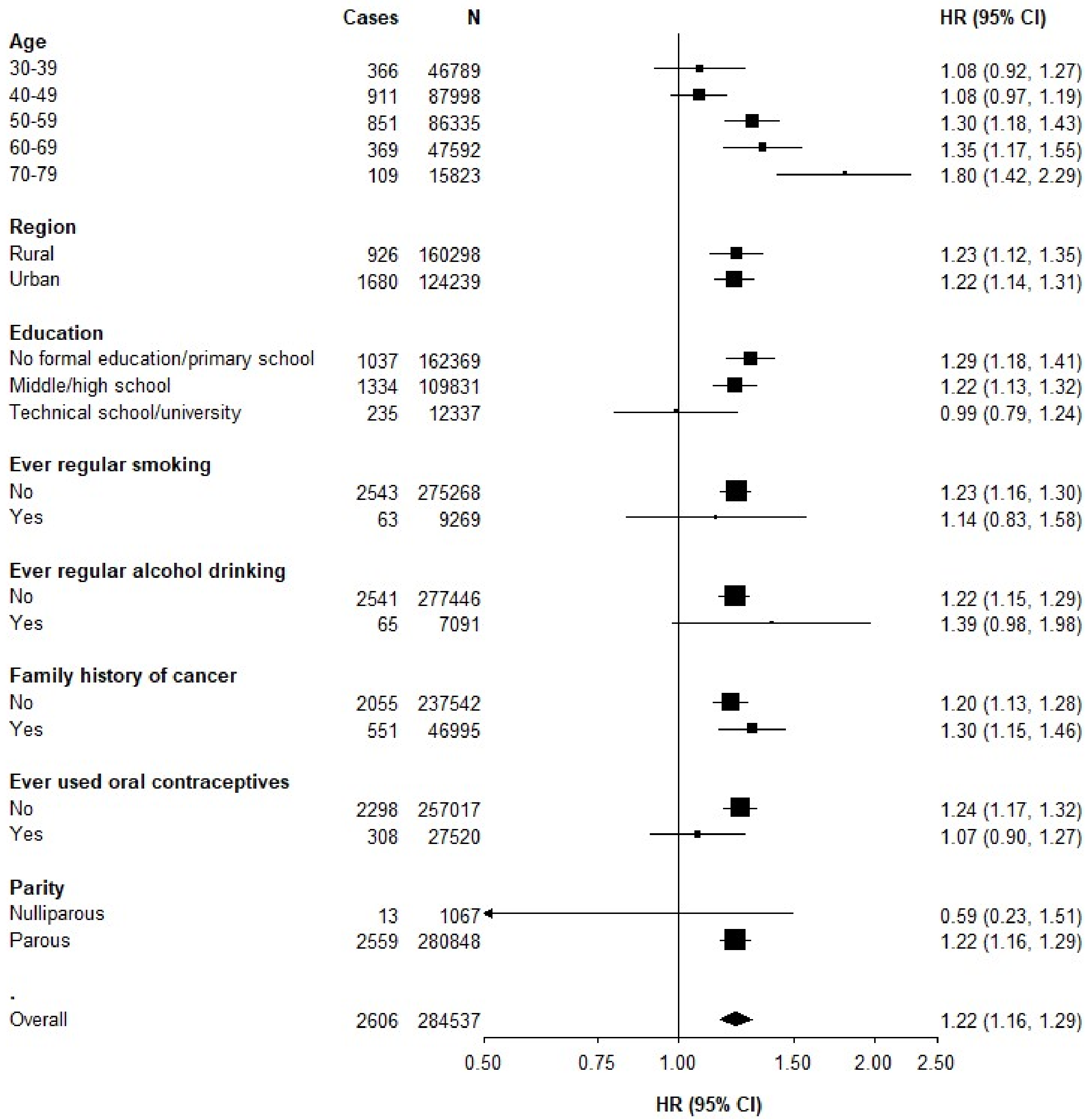

Figure S12. HRs (95% CIs) for breast cancer by waist circumference (per 10 cm) in subgroups. Adjusting for age (numeric) and menopause status (pre-, peri-, or postmenopausal) and stratifying by region.

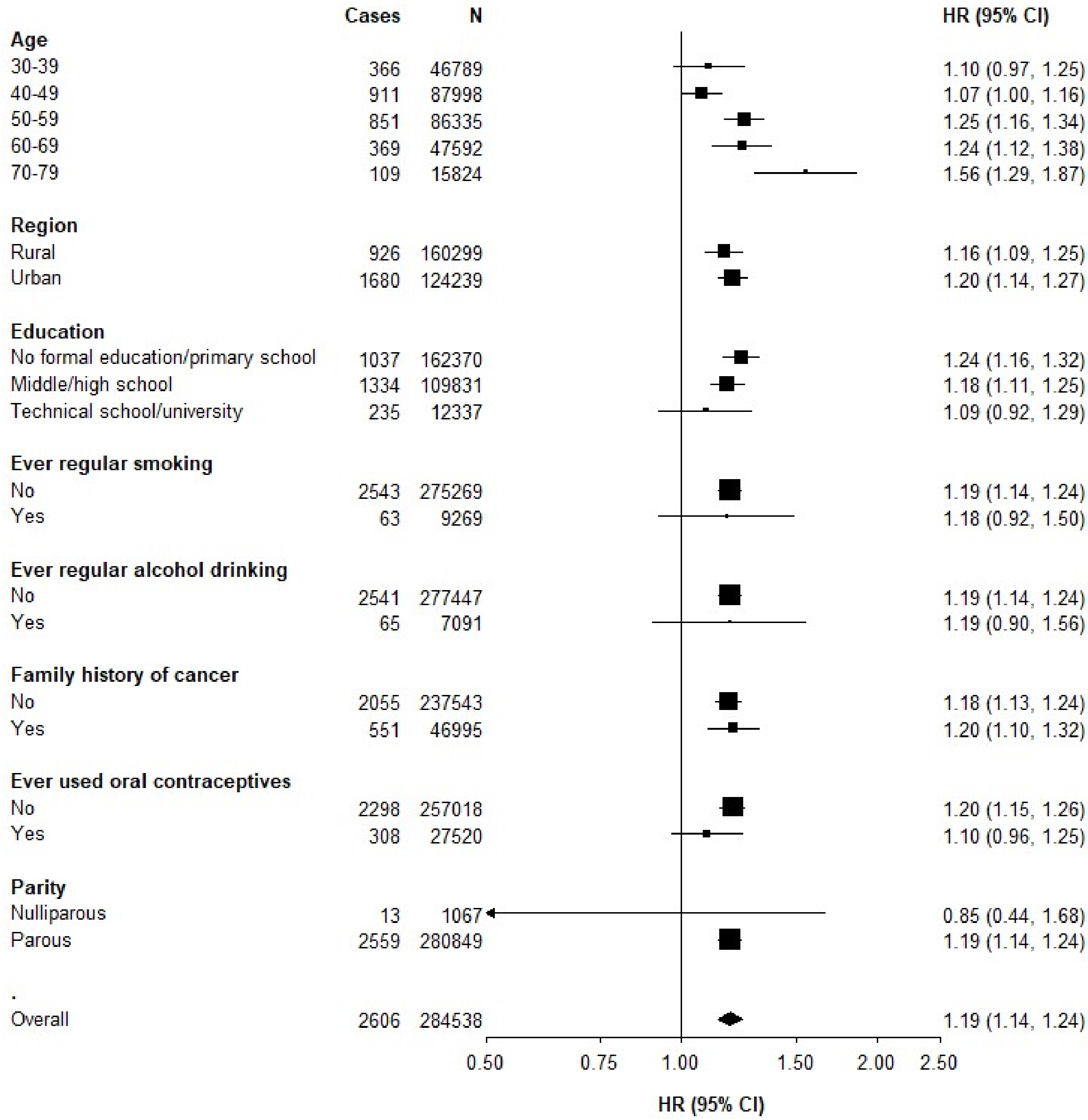

Supplement: Supplementary file 1 [file dyag155_supplementary_data.pdf]
